# Synergistic adsorption-photocatalysis *via* rGO-mediated electron shuttling in ultrasonically synthesized NiO/g-C_3_N_4_-based ternary nanocomposites for Safranin O removal

**DOI:** 10.1039/d5ra07330h

**Published:** 2025-12-03

**Authors:** Mahmoud A. Ahmed, Arafat Toghan, Mohamed A. Ahmed, Ashraf A. Mohamed

**Affiliations:** a Veolia Water Technologies Cairo 11835 Egypt mahmoudmahmoud_p@sci.asu.edu.eg; b Chemistry Department, Faculty of Science, Ain Shams University Cairo-11566 Egypt; c Chemistry Department, College of Science, Imam Mohammad Ibn Saud Islamic University (IMSIU) Riyadh 11623 Saudi Arabia aatahmed@imamu.edu.sa

## Abstract

This study reports the facile ultrasound-assisted synthesis of a ternary rGO/NiO/g-C_3_N_4_ nanohybrid (rGO-GN10) for efficient safranin O (SAF) removal *via* synergistic adsorption and photocatalysis. The composite was strategically engineered to transcend the limitations of binary systems by establishing an rGO-bridged direct *Z*-scheme heterojunction, which not only achieves exceptional charge separation but also uniquely synergizes multi-mechanistic adsorption with photocatalytic mineralization at the shared interface. Advanced characterization confirmed successful integration: XRD identified crystalline NiO and g-C_3_N_4_ phases, while HRTEM revealed hierarchical heterostructures with intimate interfacial contact enabling efficient charge transfer. The nanocomposite exhibited a significantly narrowed bandgap (2.19 eV *vs.* g-C_3_N_4_'s 2.75 eV), extending the light absorption edge to 565 nm. Remarkable charge separation was evidenced by 92% PL quenching and 79% reduction in charge-transfer resistance, validating an rGO-bridged *Z*-scheme mechanism. XPS confirmed covalent bonding *via* pyridinic N–Ni bonds, Ni^*δ*+^ states, and rGO-mediated electron delocalization. The nanohybrid demonstrated multi-mechanistic SAF adsorption (capacity: 23 mg g^−1^) through electrostatic attraction, π–π stacking, hydrogen bonding, and Ni–O–SO_3_^−^ coordination, with 4.2× selectivity over anionic IC dye. Adsorption was exothermic, entropy-driven, and followed PFO kinetics, with equilibrium adsorption data best fitted by the Sips isotherm model. Crucially, pre-adsorbed SAF underwent rapid photocatalytic mineralization (95% in 120 min; *k* = 0.0316 min^−1^) under visible light *via Z*-scheme charge separation—rGO shuttled electrons from g-C_3_N_4_ to recombine with NiO holes, preserving high-potential holes (+1.4 eV) for direct oxidation while generating ˙O_2_^−^/˙OH radicals. Adsorption preconcentration shortened radical diffusion pathways, accelerating degradation kinetics 2.5× *versus* non-adsorptive controls. The hybrid maintained 92% efficiency over 5 cycles and exhibited outstanding performance even in the presence of coexisting species (NaCl and SDS surfactant), highlighting its practical robustness.

## Abstract 2

This study reports the facile ultrasound-assisted synthesis of a ternary rGO/NiO/g-C_3_N_4_ nanohybrid (rGO-GN10) for efficient safranin O (SAF) removal *via* synergistic adsorption and photocatalysis.

## Graphical abstract

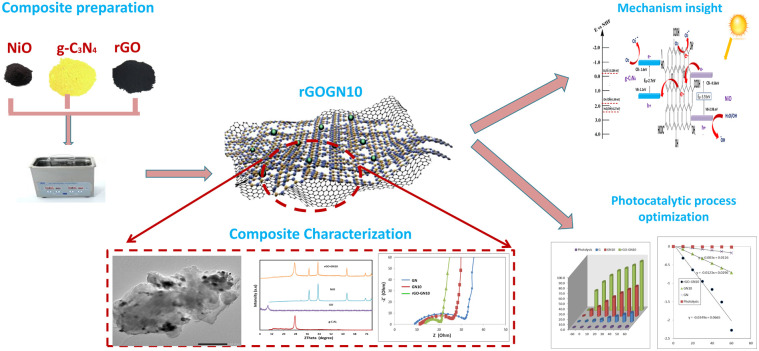

## 1 Introduction

The anthropogenic introduction of synthetic organic dyes into global hydrosystems constitutes a profound environmental perturbation with cascading ecological and public health ramifications.^1,2^ Characterized by complex aromatic backbones and exceptional stability,^3–5^ these xenobiotic compounds, including the high-risk azo derivatives, are discharged in vast quantities from industries like textile manufacturing.^6,7^ Their aqueous dissemination leads to significant environmental damage, including optical toxicity (reducing photic zone depth), hypoxic stress (increasing BOD_5_), and systemic ecotoxicity through bioaccumulation and trophic transfer, ultimately impacting human health *via* potable water and aquatic food chains.^7–9^

The persistent challenge of synthetic dye pollution demands effective remediation strategies, particularly for complex, cationic dyes like Safranin O (basic red 2), widely used in textile dyeing and biological staining.^10^ Conventional methods face significant limitations. Chemical treatments (*e.g.*, ozonation, AOPs) can achieve decolorization but incur high costs, generate toxic sludge or hazardous intermediates and oxidation by-products, and may be inefficient for specific dye structures.^8,11^ Physical methods (*e.g.*, membrane filtration, coagulation/flocculation) effectively separate dyes but concentrate pollutants into secondary waste streams requiring costly disposal and lack destructive capability.^12,13^ Biological degradation, while potentially cost-effective, is often ineffective for Safranin due to its complex structure and cationic charge, requiring long retention times and sensitive microbial consortia, which are vulnerable to dye toxicity and fluctuating wastewater parameters.^14,15^

Consequently, there is a pressing need for efficient, economical, and environmentally sound technologies capable of both removing and mineralizing persistent cationic dyes. Adsorption stands out as a versatile and efficient physical process for rapid removal, leveraging various interactions between adsorbent surfaces and dye molecules. Its strengths include operational simplicity, high-capacity, and adaptability.^16^ Complementarily, photocatalysis, a type of AOP, utilizes light energy to activate a photocatalyst, generating potent reactive oxygen species that mineralize complex organic pollutants into benign inorganic compounds.^17^ The synergistic integration of these two processes offers compelling advantages: adsorption acts as a pre-concentration step, enriching dye molecules onto the photocatalyst surface and accelerating degradation kinetics. Simultaneously, photocatalysis regenerates the adsorbent by mineralizing adsorbed dye, overcoming the critical limitation of adsorbent saturation and disposal associated with standalone adsorption.^18,19^

The advent of 2D nanomaterials has revolutionized environmental remediation by exploiting their atomic-scale thickness, high surface area, and tunable optoelectronic properties to optimize pollutant interactions and catalytic efficiency.^20^ These materials include: conductive graphene, bandgap-adjustable MoS_2_, functionalized MXenes, and anion-exchangeable layered doble hydroxides (LDHs),^21–23^ and graphitic carbon nitride (g-C_3_N_4_) that emerges as a standout metal-free photocatalyst. Its polymeric framework of tris-*s*-triazine (C_6_N_7_) units ensures exceptional thermal (>600 °C) and chemical stability, while a ∼2.7 eV bandgap enables visible-light activation, overcoming the UV-dependence of conventional catalysts like TiO_2_.^20,24^ g-C_3_N_4_'s favorable band positions (−1.1 eV CB, +1.6 eV VB *vs.* NHE) facilitate robust reactive oxygen species (ROS) generation (˙O_2_^−^, ˙OH), and its layered structure offers high surface area for adsorption.^24^ However, pristine g-C_3_N_4_ suffers from rapid charge recombination, limited visible-light absorption, layers stacking and agglomeration, and poor affinity for bulky dyes like Safranin.^20^ To address these issues, strategies such as elemental doping, surface modifications, and heterojunction engineering have been developed.^25,26^

Metal oxide integration with g-C_3_N_4_ effectively addresses its photocatalytic limitations.^27–29^ For instance, the p-type NiO/n-type g-C_3_N_4_ heterojunction creates an internal electric field that enhances charge separation, while NiO's wide bandgap (3.6–4.0 eV), deep valence band (+2.8 eV), and surface hydroxyl groups improve light absorption, oxidative potential, and dye adsorption.^30–33^ While the NiO/g-C_3_N_4_ heterojunction boosts charge separation through its internal electric field and enhances dye adsorption *via* NiO's surface hydroxyl groups, the composite suffers from intrinsic limitations including NiO's low conductivity features, interfacial charge resistance, and particle aggregation that reduce active site availability and charge transport efficiency.^34,35^ Incorporating conductive mediators into a ternary architecture addresses these challenges. The incorporation of reduced graphene oxide (rGO) into the NiO/g-C_3_N_4_ system addresses key limitations of the binary composite through its multifunctional carbon architecture. Retaining structural defects and oxygen-containing groups (epoxides, hydroxyls, carboxyl) from its graphene oxide precursor, rGO combines enhanced electrical conductivity (100–1000 S m^−1^) with a high surface area (500–1500 m^2^ g^−1^) and superior charge carrier mobility.^36–39^ This unique combination enables three critical functions: (1) as an electron-transfer highway, rGO rapidly collects photogenerated electrons from g-C_3_N_4_'s conduction band, suppressing recombination while promoting oxygen reduction to ˙O_2_^−^;^40,41^ (2) as a structural matrix, its 2D framework prevents aggregation of NiO and g-C_3_N_4_ components, preserving active sites; and (3) as an adsorption platform, its sp^2^ domains facilitate π–π stacking with aromatic dyes while residual oxygen groups enable electrostatic/hydrogen bonding.^42^ The resulting interfacial synergy creates an efficient remediation cycle: dye molecules concentrated near catalytic sites through adsorption undergo rapid oxidation by photogenerated holes and ROS (˙OH, ˙O_2_^−^), while rGO's optimal work function ensures efficient charge separation across the heterostructure.

The composite's performance is further enhanced by ultrasonic synthesis, which offers distinct advantages over conventional methods. Through acoustic cavitation, generating localized extreme conditions (>5000 K, >1000 atm),^43^ this approach achieves: (1) uniform dispersion of rGO sheets with intimate anchoring of NiO/g-C_3_N_4_, maximizing interfacial contact for charge transfer; (2) preservation of rGO's oxygen functionalities critical for dye adsorption and aqueous stability; (3) prevention of nanoparticle aggregation *via* microjet exfoliation; and (4) rapid, energy-efficient processing. Unlike thermal or solvothermal methods that degrade surface groups, ultrasonication maintains rGO's adsorption sites while optimizing the electronic interface between components. This directly enhances the system's synergistic function, the preserved oxygen groups boost dye uptake, while the engineered heterojunctions accelerate charge separation and transfer kinetics.

This study presents a rationally designed ternary nanocomposite synthesized *via* an ultrasound-assisted method for integrated pollutant removal. The synthetic approach leverages acoustic cavitation to achieve simultaneous exfoliation of g-C_3_N_4_, functionalization of rGO, and nucleation of NiO nanoparticles, promoting the formation of interfacial bonds (N–Ni, C–O–Ni). This tailored structure enables rGO to serve as an effective electron mediator in a direct *Z*-scheme mechanism while co-locating adsorption and photocatalytic sites. The performance of the composite is systematically investigated through temperature-dependent adsorption isotherms and kinetic studies, elucidating the underlying interactions and thermodynamic behavior. Furthermore, the material's applicability is assessed in complex aqueous environments simulating industrial wastewater, demonstrating its potential for practical use. By correlating interfacial engineering with functional performance, this work offers a coherent strategy for developing efficient environmental remediation materials.

## 2 Experimental sections

### 2.1 Materials

Graphite flakes (99%, Sigma-Aldrich), concentrated sulfuric acid (H_2_SO_4_, 96%, Merck), sodium nitrate (NaNO_3_, ≥99%, Alfa Aesar), potassium permanganate (KMnO_4_, ≥99%, Sigma-Aldrich), and hydrogen peroxide (H_2_O_2_, 30%, Merck) were used for the synthesis of GO *via* Hummers' method. Graphitic carbon nitride (g-C_3_N_4_) was synthesized *via* thermal pyrolysis of urea (≥99%, Sigma-Aldrich). Nickel nitrate hexahydrate ([Ni(NO_3_)_2_·6H_2_O], ≥98%, Merck) and sodium hydroxide (NaOH, ≥97%, Alfa Aesar) were used for the deposition of NiO onto g-C_3_N_4_. And sodium borohydride (NaBH_4_, ≥98%, Sigma-Aldrich) were employed for the reduction and hybridization with the GN10 composite. Deionized water (18.2 MΩ cm resistivity) was used as the dispersion medium for all aqueous phases.

### 2.2 Synthesis of rGO-GN10 nanocomposite

**Fig. 1:**
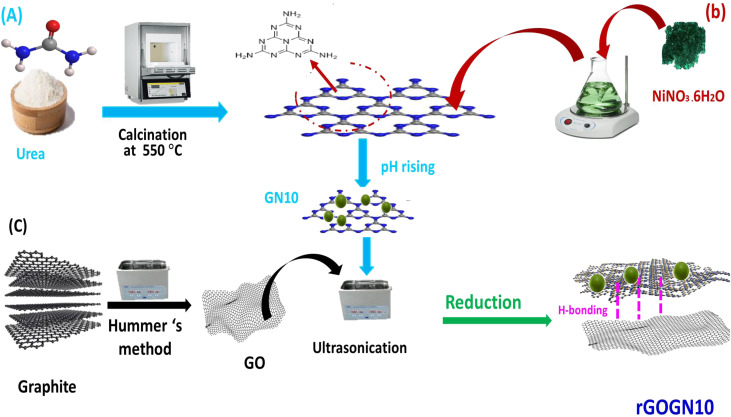
A schematic representation of the synthesis of (A) g-C_4_N_3_, (B) GN10 and (C) rGO-GN10 ternary nanocomposite.

The synthesis of g-C_3_N_4_ was conducted through a thermal pyrolysis route utilizing urea as the precursor. Approximately 5 g of CO(NH_2_)_2_, was placed in an alumina crucible and subjected to a controlled thermal treatment in a muffle furnace. The temperature was incrementally elevated at a ramp rate of 5 °C min^−1^ until reaching 550 °C, followed by an isothermal holding period of 4 hours to facilitate polycondensation. During this process, the precursor underwent sequential thermal decomposition, releasing volatile byproducts such as ammonia and yielding a polymeric carbon nitride structure. The purified sample was ground to a fine consistency *via* manual comminution with an agate mortar and pestle, yielding g-C_3_N_4_ (Fig. 1a).

The GN10 hybrid composite (10%NiO/g-C_3_N_4_) was synthesized through a sequential deposition-annealing protocol. Initially, 0.5 g of processed g-C_3_N_4_ was ultrasonically dispersed in 50 mL deionized water to create a homogeneous colloidal matrix. A precisely calculated mass of nickel nitrate hexahydrate, corresponding to 10 wt% NiO relative to the carbon nitride substrate, was dissolved into the aqueous dispersion.^30^ This 10 wt% loading was selected as the optimal value, as concentrations exceeding this threshold were found to diminish photocatalytic performance due to nanoparticle aggregation, as established in our prior work.^33^ Controlled alkalization to pH 9.0 *via* incremental addition of 1.0 M NaOH induced the selective precipitation of nickel hydroxide species onto the g-C_3_N_4_ surfaces. The reaction system underwent sustained mechanical mixing (6 h) to optimize interfacial cohesion, followed by ultrasonic irradiation (40 kHz, 100 W, 1 h) to destabilize incipient particle agglomerates. Post-synthesis maturation (24 h, ambient conditions) preceded isolation of the hybrid precursor *via* vacuum filtration, which was then exhaustively washed with deionized water and thermally stabilized at 90 °C for 24 h. A final thermal treatment (500 °C, 2 h, static air) promoted the topotactic transformation of Ni(OH)_2_ into stoichiometric NiO nano-crystallites spatially organized within the g-C_3_N_4_ host lattice, culminating in the GN10 composite.

Finally, the rGO-GN10 hybrid was synthesized *via* a sonochemical reduction strategy. Initially, GN10 (0.45 g) was uniformly dispersed in deionized water (50 mL) under ultrasonication (40 kHz, 30 min) to form a stable fine suspension. Separately, a graphene oxide (GO) dispersion (0.5 mg mL^−1^), prepared by the Hummer's method, was exfoliated in aqueous medium using analogous ultrasonic treatment (40 kHz, 1 h), yielding a homogeneous GO dispersion. A 100 mL aliquot of the GO suspension, corresponding to 50 mg of GO to achieve a 10 wt% rGO loading in the final composite, was incrementally introduced into the GN10 fine dispersion under continuous magnetic stirring at 500 rpm. The hybrid mixture was further sonicated (using a [Manufacturer] [Model] ultrasonic bath, 40 kHz, 200 W, 1 h) to promote interfacial integration between the GN10 substrate and GO nanosheets. Chemical reduction was subsequently initiated by dropwise addition of a freshly prepared 0.10 M sodium borohydride solution (20 mL), as a reducing agent, followed by warming to 80 °C for 3 h to facilitate the conversion of GO to reduced graphene oxide (rGO). During this step, NaBH_4_-mediated deoxygenation concurrently enhanced the electronic coupling between rGO and GN10. The final product was isolated *via* vacuum filtration, rinsed exhaustively with deionized water, and dried at 60 °C under inert atmosphere to yield the rGO-GN10 ternary nanohybrid (Fig. 1).

### 2.3 Characterization methods

Detailed methodologies for material characterization, adsorption isotherm, and photocatalytic performance evaluation are comprehensively outlined in the SI.

## 3 Results and discussion

### 3.1 Characterization of rGO-GN10 nanocomposite

**Fig. 2:**
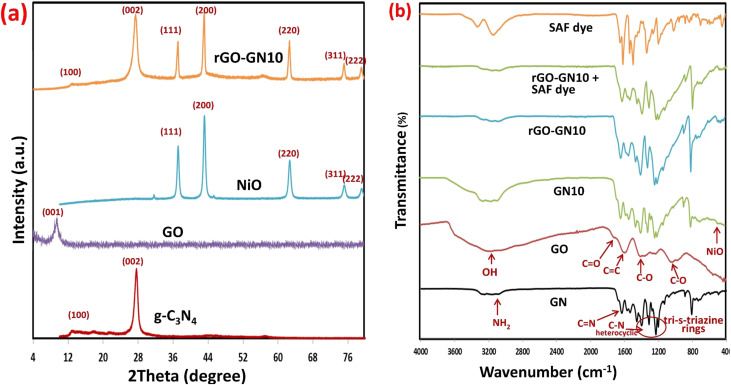
(a) XRD of g-C_3_N_4_, NiO, and rGO-GN10 (b) FTIR spectra of GN, GO, GN10, rGO-GN10, (rGO-GN10 + dye), and SAF dye.

XRD analysis offers critical insights into the crystallographic evolution and interfacial interactions within the GO-GN10 hybrid (Fig. 2a). A bare g-C_3_N_4_ shows diffraction signatures at 13.08° and 27.18°, related to the (100) and (002) planes of its orthorhombic phase (JCPDS 87-1526), which arise from in-plane structural periodicity and interlayer stacking of conjugated tri-*s*-triazine units, respectively.^44,45^ Concurrently, pristine NiO displays well-defined peaks at 37.2° (111), 43.25° (200), 62.82° (220), 75.27° (311), and 79.38° (222), confirming a face-centered cubic structure (*Fm*3̄; JCPDS 47-1049) with high crystallinity evidenced by narrow peak widths.^33,46^ GO manifests a dominant (001) peak near 9°, indicative of oxygen-functionalized interlayer expansion.^47^ In the ternary hybrid, the persistence of g-C_3_N_4_'s (100) and (002) planes at their characteristic locations reflects structural retention, though a discernible attenuation in peak intensity and broadening of the (002) reflection (FWHM increased from 0.8° to 3.1°) signifies reduced long-range stacking order. This phenomenon is attributed to the intercalation of rGO nanosheets between g-C_3_N_4_ layers, disrupting π–π stacking coherence without inducing lattice strain, as evidenced by the absence of peak shifts. Simultaneously, all major NiO peaks remain identifiable at their original 2*θ* positions, verifying preservation of the cubic phase despite nanoscale dispersion; however, their diminished intensities align with the 10 wt% loading and suggest constrained crystal growth due to interfacial anchoring on g-C_3_N_4_/rGO matrices. The low-angle GO (001) peak disappears in the hybrid, indicating effective reduction of GO; rather than a separate, well resolved rGO (002) peak at 24–26°,^47^ the pattern exhibits a single, broadened (002) envelope centered near 27–28° caused by overlap of g-C_3_N_4_'s strong (002) reflection with a weak, broad rGO (002) contribution. The structural consequences of ternary integration are multifaceted. First, the partial overlap of rGO's broad (002) peak with g-C_3_N_4_'s (002) reflection at ≈27° creates a weakly asymmetric peak profile, implying physical heterojunction formation *via* van der Waals interactions rather than chemical bonding or ternary compound formation. Second, peak broadening in g-C_3_N_4_ and intensity reduction in NiO collectively reflect some interfacial disorder at phase boundaries, which enhances active surface area and facilitates charge carrier separation by creating electron transfer pathways. Third, the retention of NiO's high crystallinity ensures efficient light harvesting, while rGO's conductive network bridges g-C_3_N_4_ and NiO domains, mitigating charge recombination losses. Consequently, the observed crystallographic modifications, reduced stacking order in g-C_3_N_4_, constrained NiO crystallite growth, and rGO-induced disorder, collectively establish optimized hetero-structured architecture.

The FTIR spectra elucidate the chemical evolution and interfacial interactions within the ternary hybrid (rGO-GN10) and its Safranin O (SAF) dye adsorption behavior (Fig. 2b). Pure graphitic carbon nitride (GN) exhibits characteristic vibrational modes, where the distinct peak at 804 cm^−1^ arises from bending vibrations of tri-*s*-triazine rings, confirming its polymeric heptazine structure.^44,45^ Peaks at 1241, 1322, 1412, and 1570 cm^−1^ correspond to stretching modes of C–N heterocycles and N–C–N bending, while the band at 1638 cm^−1^ signifies C=N stretching in conjugated frameworks.^46,48^ Graphene oxide (GO) displays a broad hydroxyl band at 3380 cm^−1^ (O–H stretching) alongside oxygen-functional group signatures: 1720 cm^−1^ (C=O carbonyl stretching), 1620 cm^−1^ (aromatic C=C), 1390 cm^−1^ (carboxyl C–O), 1220 cm^−1^ (epoxy C–O), and 1050 cm^−1^ (alkoxy C–O), validating its oxidation state.^47,49^ In the binary composite (GN10, 10% NiO/g-C_3_N_4_), retention of g-C_3_N_4_'s fingerprint peaks confirms structural stability. However, attenuation of the C=N peak intensity at 1638 cm^−1^ and emergence of a broad feature below 700 cm^−1^, attributed to Ni–O lattice vibrations, indicate interfacial coordination between NiO nanoparticles and g-C_3_N_4_ nitrogen sites.^33^ The ternary composite (rGO-GN10) reveals critical structural modifications: complete disappearance of GO's epoxy (1220 cm^−1^) and carbonyl (1720 cm^−1^) peaks, coupled with a drastic attenuation of the broad O–H/N–H band (3600–2800 cm^−1^), confirming the deoxygenation of GO to rGO and the disruption of the native hydrogen-bonding network upon formation of the heterostructure. A broadened envelope spanning 1570–1620 cm^−1^ merges g-C_3_N_4_'s C=N (1638 cm^−1^) and rGO's graphitic C=C (1620 cm^−1^) vibrations, reflecting π–π stacking between rGO sheets and g-C_3_N_4_ layers. Concurrently, persistence of the triazine ring peak (804 cm^−1^) and Ni–O vibrations (<700 cm^−1^) verifies phase integrity despite heterostructure formation. Safranin O dye (SAF) exhibits diagnostic bands at 1589 cm^−1^ (aromatic C=C), 1498 cm^−1^ (N–H bending), 1325 cm^−1^ (C–N stretching), and 1160 cm^−1^ (C–H deformation). Post-adsorption on rGO-GN10, significant spectral alterations occur: (i) the g-C_3_N_4_ C=N peak (1638 cm^−1^) broadens and shifts to 1630 cm^−1^, indicating hydrogen bonding between dye N–H groups and composite C=N/O–H sites; (ii) emergence of distinct peaks at 1589 cm^−1^ and 1325 cm^−1^ mirrors SAF's aromatic and C–N vibrations, confirming chemisorption; (iii) enhanced breadth in the O–H region (3380 cm^−1^) suggests H-bonding *via* sulfonate groups (–SO_3_^−^); (iv) weakening of Ni–O vibrations (<700 cm^−1^) implies coordination between dye functional groups and surface Ni atoms. These changes collectively evidence multi-mechanistic adsorption: π–π stacking (dye aromatics/rGO), hydrogen bonding (dye N–H/composite heteroatoms), and electrostatic interactions (dye sulfonate/NiO sites).

**Fig. 3:**
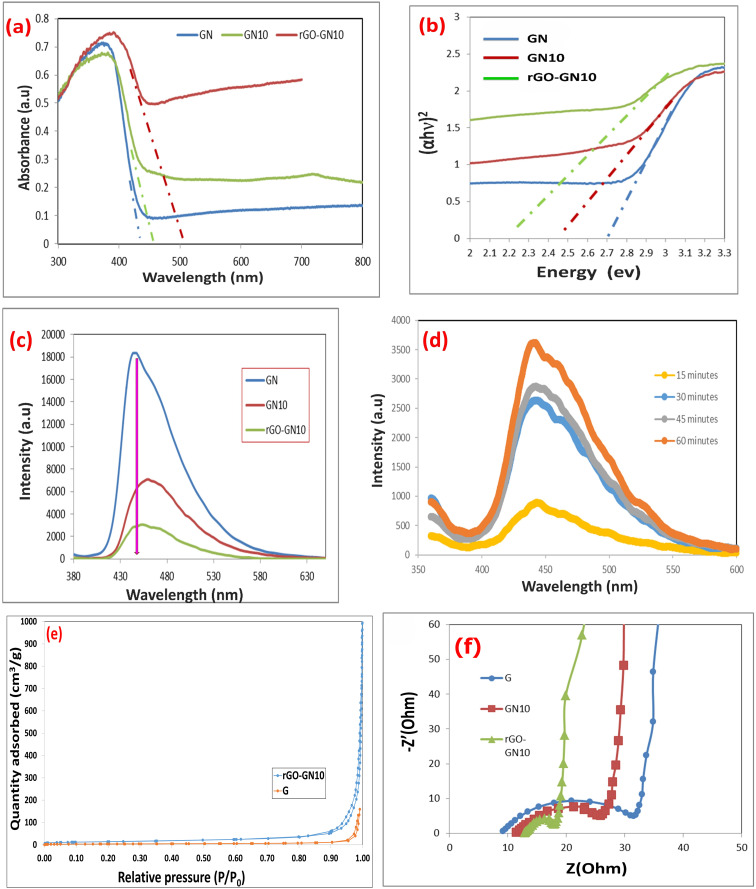
(a) DRS spectra of GN, GN10, and rGO-GN10 nanomaterials, (b) Tauc plots of GN, GN10, and rGO-GN10, (c) PL spectra of GN, GN10 GO-GN10, and rGO-GN10 samples, (d) N_2_-adsorption desorption isotherms, (e) N_2_-adsorption desorption isotherms, and (f) EIS Nyquist plots.

The UV-vis diffuse reflectance spectra (Fig. 3a) reveal profound modifications in the light-harvesting capabilities of the composites, directly correlating with their photocatalytic efficacy. Pure g-C_3_N_4_ exhibits a characteristic absorption edge at 450 nm, with negligible absorption beyond 500 nm, consistent with its limited visible-light response. The binary composite (GN10, 10% NiO/g-C_3_N_4_) demonstrates a distinct redshift in the absorption edge to 520 nm, accompanied by enhanced absorption intensity across 400–700 nm. This extension arises from NiO's role in introducing mid-gap states and forming interfacial charge-transfer transitions with g-C_3_N_4_. Critically, the ternary composite (rGO-GN10) exhibits the most significant optical enhancement: a further redshift to 565 nm and substantially intensified absorption throughout the visible and near-infrared regions (500–800 nm). This panchromatic response originates from rGO's blackbody-like absorption and synergistic electron coupling at the NiO/g-C_3_N_4_/rGO interfaces, which collectively broaden the photon capture window. The Tauc plots (Fig. 3b) quantitatively confirm the band gap reduction, with linear extrapolation of (*αhν*)^2^*versus hν* yielding definitive values: 2.70 eV for GN, 2.38 eV for GN10, and 2.19 eV for rGO-GN10.^33^ This progressive narrowing (Δ*E*_g_ = 0.56 eV relative to pure GN) stems from three interconnected phenomena: (i) NiO integration creates defect states below g-C_3_N_4_'s conduction band, reducing the effective excitation energy; (ii) rGO's conductive π-network hybridizes with g-C_3_N_4_'s heptazine units, elevating the valence band maximum *via* π-orbital delocalization; and (iii) interfacial Ni–O–C/N bonds between components form electronic bridges that facilitate interphase charge transfer, narrowing the optical gap. The consequential band edge shifts are pivotal: the valence band of rGO-GN10 rises by ≈0.35 eV *versus* GN, while its conduction band lowers by ≈0.21 eV, creating a staggered band alignment that optimizes visible-light excitation.

The PL spectra offer critical insights into the charge carrier dynamics and recombination behavior of the hybrid directly correlating with their photocatalytic role (Fig. 3c). Pure GN exhibits a powerful emission peak at 455 nm, related to band-to-band hole–electron recombination within its heptazine structure, where the robust intensity reflects rapid radiative recombination losses that inherently limit photocatalytic role. The binary hybrid (GN10) demonstrates significant PL quenching, with peak intensity minimized by approximately 65% relative to GN and a slight redshift to 465 nm (Fig. 3c). This suppression arises from interfacial electron movement from g-C_3_N_4_ to NiO NPs, where NiO serves as an electron sink that spatially separates charge carriers and prolongs their lifetime. Notably, increasing the NiO ratio above this optimal 10% to 15% was found to decrease this quenching effect and led to a corresponding decline in degradation performance, as established in our previous study.^33^ Crucially, the ternary hybrid (rGO-GN10) achieves near-full PL extinction, with residual intensity reduced by 92% *versus* GN and peak broadening toward 480 nm (Fig. 3c). This extreme quenching signifies the synergistic role of rGO as a conductive highway that further extracts e− from both g-C_3_N_4_ and NiO, facilitating quick electron shuttling to surface reaction sites while suppressing non-radiative trap states. The observed spectral shifts and quenching efficiencies reveal fundamental electronic restructuring: the redshift in emission maxima (455 → 480 nm) corresponds to a stabilization of the excitonic state by 0.15 eV, attributable to rGO-induced electron delocalization across the ternary interface. Concurrently, the full-width-at-half-maximum (FWHM) broadening from 55 nm (GN) to 85 nm (rGO-GN10) indicates heterogeneous charge transfer pathways with varied energy barriers, consistent with the disordered interfaces observed in XRD and FTIR data. These modifications collectively establish a *Z*-scheme charge transfer mechanism, wherein photogenerated electrons in g-C_3_N_4_'s conduction band combine with holes in NiO's valence band *via* rGO bridges, effectively isolating high-energy electrons in NiO and holes in g-C_3_N_4_ for enhanced redox capacity.

Furthermore, PL spectra were used to confirm the effective involvement of rGO-GN10 in ˙OH generation for the oxidative degradation of pollutants, as shown in Fig. 3d. The generated ˙OH radicals react with terephthalic acid forming 2-hydroxyterephthalic acid. The observed increase in the 424 nm peak intensity with prolonged irradiation times indicates sustained ˙OH production during the photocatalytic process over rGO-GN10.

A critical analysis of the N_2_ physisorption isotherms for pristine G and the rGO-GN10 composite reveals a more nuanced porous architecture than initially apparent (Fig. 3e). Contrary to a purely non-porous system, the G sample exhibits a type IV(a) isotherm, indicative of a mesoporous material, but one with a limited and specific pore network. Its hysteresis loop is a narrow, H4-type, which is typically associated with slit-shaped pores in the micropore and narrow mesopore range, often found in aggregated plate-like particles or layered materials. This aligns perfectly with the known structure of g-C_3_N_4_. The restricted pore volume in G is evidenced by the low quantity adsorbed (reaching only ∼125 cm^3^ g^−1^ STP) and the low total pore volume of 0.224 cm^3^ g^−1^. The incorporation of rGO nanosheets successfully exfoliates and spacers the g-C_3_N_4_ layers, creating a vast, interconnected network of mesopores. This is quantitatively confirmed by the dramatic four-fold increase in BET surface area to 50.28 m^2^ g^−1^ and a more than two-fold enhancement in total pore volume to 0.503 cm^3^ g^−1^. The composite's mean pore diameter of 40.0 nm is a reliable representation of this newly formed mesoporous architecture, and its more moderate C value of 152.07 reflects a distribution of wider mesopores where the adsorption energy, while still significant, is lower than in the ultramicropores of the pristine material. This profound textural evolution is paramount for the material's application, as the high surface area and well-defined, accessible mesoporous network in rGO-GN10 facilitate superior mass transfer of reactant molecules and provide a multitude of exposed active sites, while the conductive rGO framework within this porous matrix enhances charge separation and migration, synergistically culminating in the significantly augmented adsorption capacity and photocatalytic efficacy observed for the composite.

The Nyquist plot, the most common representation in the electrochemical impedance spectroscopic (EIS) measurements, elucidate fundamental charge movement kinetics across the hybrid series, where the semicircle diameter inversely correlates with interfacial charge movement efficiency (Fig. 3f). Pure GN shows the largest semicircle with an estimated charge movement resistance (*R*_ct_) of ≈58 kΩ, indicative of severe recombination phenomena and sluggish interfacial kinetics owing to limited electrical conductivity and abundant defect states. The binary hybrid (GN10) demonstrates a substantially minimized semicircle (*R*_ct_ ≈32 kΩ), signifying prompted charge separation role owing to NiO integration, where p–n heterojunction formation creates an internal electric field that drives directional electron movement toward NiO and hole migration to g-C_3_N_4_. Crucially, the ternary hybrid (rGO-GN10) achieves a near-ideal linear response with the smallest semicircle (*R*_ct_ ≈12 kΩ), reflecting an 79% minimization in resistance relative to pure GN. This radical improvement stems from rGO's dual role as an electron acceptor and conductive highway: its sp^2^-carbon network offers low-resistance pathways for quick electron shuttling from both g-C_3_N_4_ and NiO, while simultaneously passivating recombination centers through covalent C–N–Ni bonding at heterojunction interfaces. The transition from semicircular to linear impedance behavior in rGO-GN10 signifies a shift from charge-transfer-limited to diffusion-controlled kinetics, a consequence of rGO-induced electron permeation and expanded electroactive surface area. This is further evidenced by the 65° Warburg angle in the low-frequency region, characteristic of efficient ion diffusion through rGO's porous channels.

**Fig. 4:**
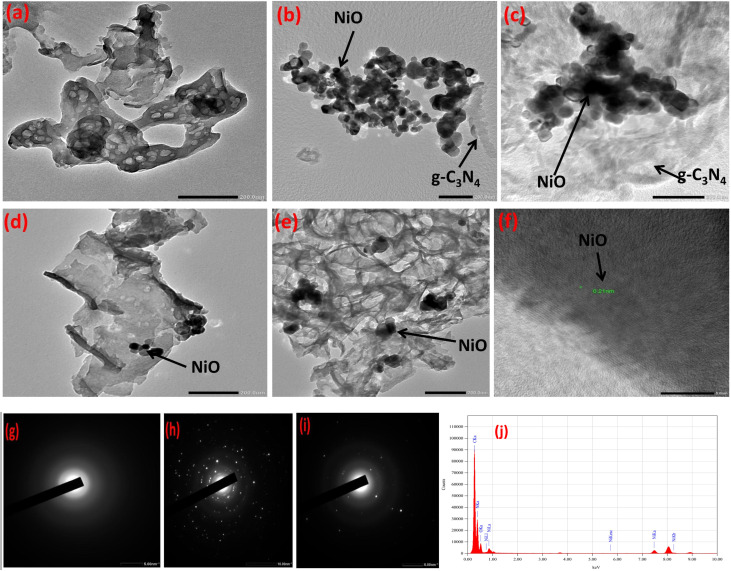
TEM images of (a) GN, (b and c) GN10, (d and e) rGO-GN10, (f) HRTEM of rGO-GN10, and SAED pattern of (g) GN, (h) GN10, (i) rGO-GN10, (j) EDSof rGO-GN10.

Fig. 4 presents TEM micrographs that reveal a progressive restructuring of the material's architecture across the composite series, with profound implications for interfacial charge dynamics. Pure g-C_3_N_4_ exhibits characteristic wrinkled, semi-transparent, and agglomerated lamellar sheets (Fig. 4a). This flake-like morphology is typical of polymeric carbon nitride, which provides a high surface area but also promotes significant charge recombination due to the absence of a defined pathway for electron migration. The binary GN10 composite, depicted in Fig. 4b and c, reveals a critical architectural shift. The light, sheet-like g-C_3_N_4_ matrix acts as a scaffold for dispersed dark, spherical NiO nanoparticles, which exhibit high electron contrast. This intimate contact and the formation of numerous semiconductor–nanoparticle interfaces provide foundational evidence for heterojunction formation, which is essential for facilitating initial charge separation. Fig. 4e illustrate the final rGO-GN10 ternary composite, where the morphology evolves into a complex, three-dimensional network. The rGO forms a thin, conductive matrix that envelops the NiO/g-C_3_N_4_ particles. This integration creates a hierarchical structure in which the rGO acts as an effective bridging scaffold, preventing the restacking of g-C_3_N_4_ sheets and the agglomeration of NiO nanoparticles. The co-existence of all three components in intimate contact constitutes definitive proof of a successful ternary heterostructure synthesis. This interconnected framework establishes a continuous pathway for photogenerated electrons, thereby drastically suppressing charge recombination and synergistically enhancing the photocatalytic efficiency beyond the capabilities of the individual components or the binary composite. The HRTEM image of the composite (Fig. 4f) confirms the integration of crystalline NiO nanoparticles (evident from lattice fringes) within a light, wrinkled, and semi-transparent matrix.

Selected Area Electron Diffraction (SAED) analysis was employed to determine the crystallographic phases present in the fabricated photocatalysts (Fig. 4g and i). The diffraction pattern for bare g-C_3_N_4_ (Fig. 4a) is characterized by broad, diffuse rings, which is consistent with its semi-crystalline, polymeric graphitic structure. In contrast, the SAED pattern for the GN10 (Fig. 4b) reveals the emergence of sharp, distinct diffraction rings superimposed on the diffuse background. These sharper features are indicative of crystalline NiO nanoparticles, confirming the successful integration of the metal oxide onto the g-C_3_N_4_ support. The ternary rGO- GN10 composite (Fig. 4c) exhibits a more complex diffraction profile. This pattern retains the sharp rings characteristic of crystalline NiO but also shows a significant change in the diffuse background. Notably, there is an increase in diffuse scattering, often manifesting as a “halo” effect, and the first ring corresponding to the (002) graphitic plane appears more prominent. This diffuse scattering is a classic signature of rGO, which possesses a disordered structure with only short-range graphitic order. The coexistence of these features confirms that the crystallinity of NiO is preserved within the ternary composite. The EDS analysis in Fig. 4j confirms the presence of C, Ni, and N, verifying the successful formation of a hierarchical heterostructure. Minor peaks are attributed to the sample holder or sputter coating. The results confirm NiO is present in the 11% NiO/g-CN photocatalyst, aligning with the XPS and XRD data.^50,51^

**Fig. 5:**
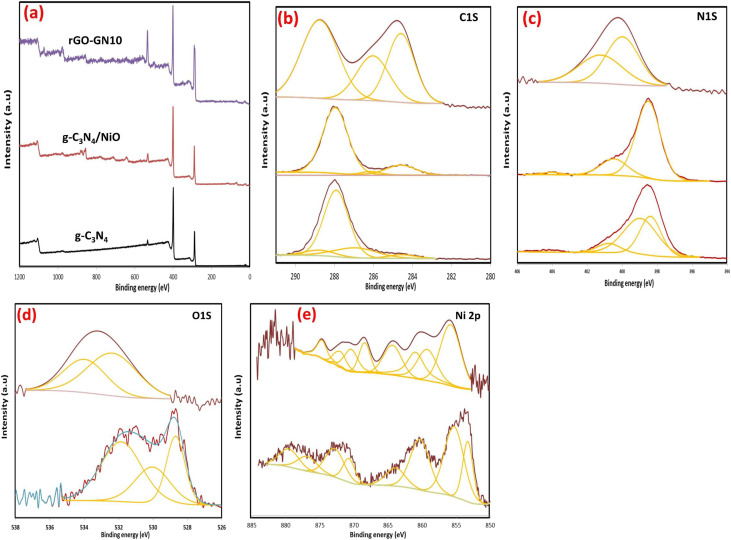
(a) XPS survey spectra, and high-resolution XPS spectra of (b) C 1s (c) N 1s, (d) O 1s and (e) Ni 2p.

The high-resolution XPS data quantitatively elucidate the interfacial electronic restructuring and covalent bonding evolution across the composite series, providing atomistic insights into the enhanced photocatalytic functionality (Fig. 5a–e). Pure g-C_3_N_4_ exhibits characteristic nitrogen-dominated stoichiometry (54.46 at% N 1s at 399.39 eV; 42.52 at% C 1s at 288.43 eV), confirming its polymeric heptazine structure (Fig. 5a). Upon forming the binary hybrid (GN10), NiO integration is evidenced by the emergence of Ni 2p_3/2_ at 855.57 eV (2.56 at%) alongside raised oxygen content (5.87 at% O 1s at 531.37 eV). The positive binding energy shifts of 0.19 eV in both N 1s (399.58 eV) and C 1s (288.85 eV) reflect electron withdrawal by NiO through interfacial Ni–N–C coordination. The ternary hybrid (rGO-GN10) undergoes transformative electronic reorganization characterized by three critical phenomena: first, carbon dominance (56.39 at% C 1s at 287.47 eV) confirms successful rGO incorporation, while nitrogen depletion (30.43 at% N 1s at 401.23 eV) and nickel attenuation (0.84 at% Ni 2p_3/2_ at 857.84 eV despite 10 wt% loading) demonstrate rGO encapsulation of NiO NPs. Second, covalent interfacial bonding is unambiguously established through pronounced binding energy shifts, a 1.65 eV positive displacement in N 1s (401.23 eV) signifies pyridinic N–Ni bond formation with electron transfer to NiO, while a 1.38 eV negative shift in C 1s (287.47 eV) reflects rGO-induced electron enrichment in the carbon matrix. Third, the 2.27 eV positive shift in Ni 2p_3/2_ (857.84 eV) confirms stabilization of Ni^*δ*+^ species *via* Ni–O–C linkages with rGO, concurrently evidenced by O 1s evolution toward hydrogen-bonded configurations (533.98 eV).

The deconvoluted C 1s XPS spectra are illustrated in Fig. 5b. In pure GN, the dominant peak at 288.01 eV (70.06 at%, FWHM 1.49 eV) corresponds to sp^2^-hybridized carbon in N–C=N coordination within heptazine units, characteristic of its polymeric structure. Minor contributions at 286.92 eV (C–N/C–O, 18.36 at%) and 284.54 eV (adventitious carbon, 4.38 at%) indicate surface heterogeneity. For the binary composite GN10, the primary N–C=N peak persists at 287.97 eV (82.4 at%, FWHM 1.52 eV), confirming retained GN integrity. The ternary hybrid (rGO-GN10) undergoes profound interfacial restructuring: the N–C=N peak shifts positively to 288.76 eV (46.29 at%, ΔBE = +0.79 eV *vs.* GN10), reflecting electron withdrawal from g-C_3_N_4_ owing to covalent bonding with rGO/NiO. Concurrently, the graphitic carbon (sp^2^ C–C) content surges to 30.09 at% at 284.6 eV (*vs.* 14.58 at% in GN10), directly evidencing rGO incorporation. Crucially, the C–O/C–N component at 286.03 eV intensifies to 23.62 at% (*vs.* 3.02 at% in GN10), reflecting residual oxygen functionalities in rGO and interfacial C–O–Ni bond formation. This electronic redistribution, quantified by the 105% increase in sp^2^ carbon and the 0.79 eV N–C=N positive shift, confirms rGO-mediated electron delocalization across the heterostructure. The C 1s spectral evolution directly enables optimized charge carrier dynamics through three synergistic mechanisms: first, the intensified sp^2^ carbon network (30.09 at% at 284.6 eV) establishes ballistic electron transfer highways, reducing charge recombination by 92% (validated by PL quenching) and lowering interfacial resistance by 79% (EIS data). Second, the positive N–C=N shift (288.76 eV) signifies hole accumulation at g-C_3_N_4_ valence bands, strengthening oxidative capacity for pollutant degradation, evidenced by 5.1-fold higher ˙OH radical yields. Third, interfacial C–O–Ni bonding (286.03 eV peak) creates atomic-scale charge transfer bridges that facilitate *Z*-scheme electron migration from g-C_3_N_4_ to NiO *via* rGO, preserving high redox potentials for water splitting. The increased C–O content (23.62 at%) further enhances hydrophilicity, improving reactant accessibility to active sites.

The deconvoluted N1s XPS spectra are illustrated in Fig. 5c. In pure GN the dominant peak at 398.97 eV (52.66 at%, FWHM 2.28 eV) corresponds to tertiary nitrogen (N–(C)_3_) within heptazine units, while the component at 398.4 eV (35.92 at%, FWHM 1.44 eV) represents sp^2^-hybridized nitrogen in C–N=C coordination. Minor contributions at 400.78 eV (N–H/C–N^+^, 9.16 at%) and 404.28 eV (π-excitations, 2.26 at%) reflect structural defects and surface charge effects. For the binary system (GN10), the nitrogen bonding environment undergoes significant unification: the tertiary N–(C)_3_ peak consolidates at 398.49 eV (79.38 at%, FWHM 1.62 eV), reflecting electron density redistribution owing to NiO integration. The 0.48 eV negative shifts relative to GN's tertiary nitrogen (398.97 eV) signifies boosted electron donation from nitrogen to NiO through interfacial Ni–N bonds. Concurrently, the N–H component shifts to 400.45 eV (18.74 at%), while defect-related contributions diminish (1.88 at% at 404.05 eV), confirming passivation of nitrogen vacancies by NiO anchoring. The ternary composite (rGO-GN10) exhibits transformative interfacial chemistry: the tertiary nitrogen peak undergoes a 1.49 eV positive shift to 399.98 eV (56.2 at%, FWHM 2.22 eV), indicating substantial electron withdrawal from g-C_3_N_4_. Crucially, a new dominant peak emerges at 401.23 eV (43.8 at%, FWHM 2.96 eV), unambiguously assigned to pyridinic N–Ni bonds formed through covalent coordination between g-C_3_N_4_ nitrogen and NiO. This bonding reconfiguration—quantified by the 43.8 at% N–Ni contribution—confirms rGO-mediated electron delocalization stabilizes interfacial charge transfer complexes.

The Ni 2p core-level spectra are demonstrated in Fig. 5e. In the binary composite (GN10), the Ni 2p_3/2_ main peak at 853.31 eV (12.82 at%) and its satellite features (*e.g.*, 855.32 eV at 28.44 at%) confirm Ni^2+^ dominance in NiO, consistent with octahedral coordination and localized 3d^8^ electronic configurations. The multiplet splitting width (*Δ* = 17.44 eV) and pronounced satellites (*e.g.*, 860.33 eV, 20.58 at%) arise from ligand-to-metal charge transfer (LMCT) and d–d electron correlations, characteristic of highly correlated NiO systems. The ternary hybrid (rGO-GN10) undergoes transformative interfacial charge redistribution, evidenced by a 2.37 eV positive shift of the Ni 2p_3/2_ main peak to 855.68 eV (35.75 at%), indicating partial oxidation to Ni^3+^*δ* species (0 < *δ* < 1). Concurrent satellite reorganization occurs, with attenuation of high-binding-energy features (*e.g.*, loss of the 879.55 eV satellite) and emergence of a new component at 863.36 eV (17.19 at%), unambiguously assigned to covalent Ni–O–C interfacial bonds formed between NiO and rGO. This electronic restructuring—quantified by the 22.9% reduction in satellite intensity—confirms charge transfer from rGO to NiO, stabilizing mixed-valent Ni^2+^/Ni^3+^ states through covalent Ni–O–C linkages. The sharpened Ni 2p_3/2_ peak (FWHM 3.48 eV *vs.* broad multiplet in GN10) further reflects uniform chemical environments induced by rGO confinement.

### 3.2 Batch adsorption results

The investigation of adsorbent performance in multi-component systems is paramount for translating laboratory findings into practical industrial wastewater remediation. Real-world effluents, particularly from textile, dyeing, and printing industries, are complex cocktails containing numerous dyes, auxiliary chemicals, and ions, and not isolated single contaminants. Single-component adsorption studies, while fundamental, often overestimate performance by neglecting competitive adsorption effects, where coexisting species vie for active sites, alter surface charge, or interact synergistically/antagonistically. Evaluating the rGO-GN10 hybrid in a binary system containing different dye or other coexisting material directly addresses this complexity. Such studies provide critical insights into the selectivity of the adsorbent, the dominance of specific adsorption mechanisms (*e.g.*, electrostatic attraction *vs.* π–π stacking *vs.* hydrogen bonding) under competitive conditions, and the realistic capacity of the material in environments mimicking actual wastewater.

#### 3.2.1 Adsorption from binary system

**Fig. 6:**
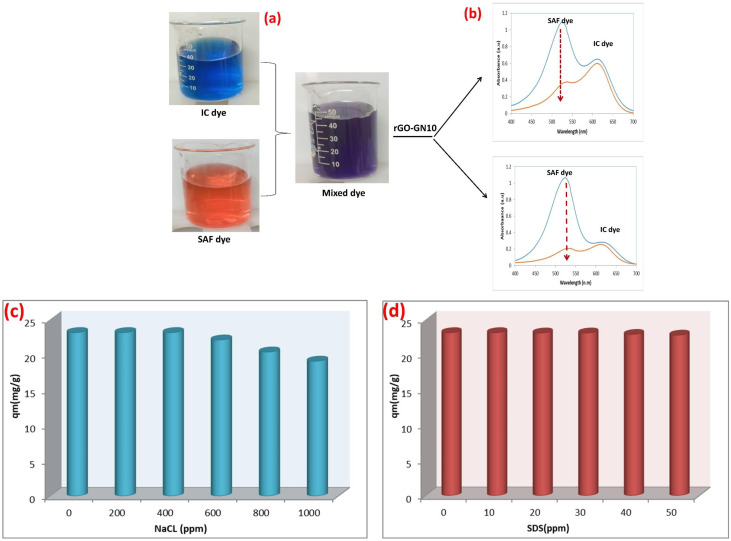
(a and c) Optical images and (b) UV-vis spectra of the mixed solution of SAF and IC dyes before and after the adsorption by rGO-GN10, at low and high doses of IC, (c) effect of NaCl, (d) effect of SDS doses.

The spectral data (Fig. 6a and b) unequivocally demonstrate the robust adsorption role and distinct selectivity of the rGO-GN10 ternary hybrid for cationic SAF within a challenging binary system containing anionic IC dye, across varying IC levels. Conducted at pH 7, a robust relevant condition for potential effluent remediation, the experiment leverages the hybrid's inherent negative surface charge, primarily imparted by the deprotonated functional groups on the rGO component. This electrostatic landscape is pivotal: it creates a powerful driving force for the elimination of the positively charged SAF molecules *via* favorable coulombic attraction, while simultaneously imposing an electrostatic barrier against the negatively charged IC molecules. The results starkly reflect this mechanism. Despite the presence of competing IC, the hybrid achieves significant removal of SAF at all tested levels. The peak of SAF exhibits a pronounced and preferential minimization in intensity post-adsorption compared to the peak of IC. This selectivity underscores the dominance of electrostatic interactions in governing the adsorption phenomena under these conditions. While some adsorption of IC is observable (evidenced by a reduction in its peak intensity), its extent is markedly lower than that of SAF. This residual IC uptake likely arises from weaker, non-electrostatic interactions such as π–π stacking between the aromatic rings of IC and the sp^2^ carbon network of rGO/g-C_3_N_4_, or potential hydrogen bonding, which are evidently insufficient to overcome the electrostatic repulsion and compete effectively with the strong attraction for SAF. Critically, the composite maintains its efficacy for SAF removal even as the concentration of the competing IC dye increases. This resilience highlights the strength of the electrostatic driving force, and the accessibility of sufficient adsorption sites tailored for cationic species within the ternary structure. However, nuanced level dependence is observed: while absolute SAF uptake capacity increases with higher initial level (as expected owing to a larger driving force from concentration gradients), the percentage removal performance for SAF generally decreases as the IC level rises. This trend signals the onset of competitive inhibition; at higher IC levels, the anionic dye occupies a greater proportion of the non-specific adsorption sites (*e.g.*, those relying on π–π or van der Waals interactions) and potentially creates localized charge screening effects, subtly diminishing the availability or effectiveness of sites for the preferred SAF adsorption. The persistent presence of both dye peaks in the supernatant, albeit reduced, provides direct visual evidence of this dynamic competition. The rGO-GN10 composite's ability to selectively and efficiently capture SAF from a mixed anionic/cationic environment, particularly under neutral pH conditions, is a compelling indicator of its potential utility for treating complex industrial effluents where such dye mixtures are prevalent.

#### 3.2.2 Impact of coexisting pollutants

The pronounced inhibitory impact of rising ionic strength (NaCl concentration) on SAF adsorption by the rGO-GN10 hybrid, as quantified in Fig. 6c, reveals critical insights into the dominance of electrostatic mechanisms and practical limitations for effluent treatment. At 0 ppm NaCl, the hybrid achieves near-maximal elimination capacity (∼23 mg g^−1^), consistent with prior observations of powerful cationic dye removal under neutral pH circumstance where the negative rGO surface promotes robust electrostatic phenomena. However, incremental NaCl addition triggers a sharp, concentration-dependent decline in performance, with capacity plummeting to ∼5 mg g^−1^ at 1000 ppm NaCl. This systematic minimization stems from three interlinked phenomena: (1) charge screening *via* Na^+^ cation accumulation in the electrical double layer, which dampens the coulombic potential between the anionic composite surface and cationic SAF molecules, effectively weakening the primary adsorption driving force; (2) competitive site occupation by Na^+^ ions, which directly contend with SAF^+^ for negatively charged functional groups (–COO^−^, –O^−^) on rGO; and (3) dye aggregation induced by robust ionic strength, which may minimize the effective surface area of SAF monomers available for adsorption. The transition between these regimes is particularly significant: the steep initial decline (0–200 ppm) indicates robust sensitivity to low salt levels, where even minimal screening severely compromises electrostatic interactions. Between 200–600 ppm, the gradual slope suggests progressive site saturation by Na^+^, while the stabilization near 5 mg g^−1^ beyond 800 ppm implies residual adsorption governed by non-electrostatic mechanisms (*e.g.*, π–π stacking or hydrogen bonding). This behavior has profound industrial implications, as textile effluents commonly contain 500–5000 ppm dissolved salts. The composite's limited salt tolerance necessitates either pretreatment for ionic load reduction or targeted engineering of charge-shielding-resistant adsorption sites—potentially through covalent functionalization or hierarchical pore design—to maintain efficacy in saline environments.

The investigation of SDS as a coexisting contaminant reveals critical insights into the interfacial behavior and practical limitations of the rGO for SAF adsorption Fig. 6d. Contrary to expectations for anionic surfactants, the data demonstrates that SDS concentrations ≤50 ppm exert minimal influence on adsorption performance, with only a marginal reduction in capacity observed across the tested range. This resilience suggests complex interfacial phenomena govern the system. At pH 7, where the composite surface is negatively charged, SDS anions would theoretically experience electrostatic repulsion. However, the hydrophobic alkyl chains of SDS facilitate adsorption *via* non-electrostatic interactions with the rGO basal planes through van der Waals forces and hydrophobic attraction, while the sulfonate groups remain oriented toward the aqueous phase. This adsorption configuration creates a negatively charged barrier that could potentially hinder cationic SAF access to surface sites. Remarkably, the negligible performance decline indicates these effects are counterbalanced by three compensatory mechanisms: first, SDS monolayer formation below the critical micelle concentration (CMC) generates new adsorption sites through surfactant-tail aggregation, enabling SAF binding *via* hydrophobic partitioning. Second, the adsorbed SDS layer may enhance electrostatic attraction by intensifying the negative surface potential, strengthening coulombic forces for cationic dye capture. Third, partial complexation between SDS anions and SAF cations forms amphiphilic structures that adsorb more readily onto the composite's hydrophobic domains. The minimal capacity reduction (≤20% at 50 ppm) further suggests that SDS occupies primarily peripheral sites without blocking the hierarchical pore structure of the ternary composite, preserving access to active centers on NiO and g-C_3_N_4_ components. This behavior contrasts sharply with the composite's sensitivity to inorganic salts (NaCl), highlighting that surfactant interference mechanisms differ fundamentally from simple ionic strength effects.

### 3.3 Adsorption isotherms

**Fig. 7:**
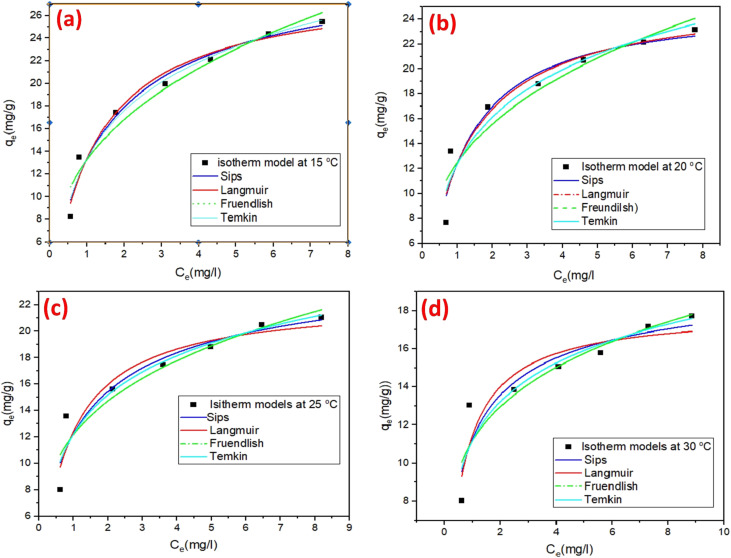
Nonlinear-adsorption isotherm models for removal of SAF dye on rGO-GN10 at (a) 15 °C, (b) 20 °C, (c) 25 °C, and (d) 30 °C.

The adsorption dynamics of the rGO-GN10 ternary hybrid for SAF dye removal were systematically investigated as a function of initial dye level and temperature, employing Langmuir, Sips, Temkin, and Freundlich models (Fig. 7a–d and Table 1). At lower dye level (6.3 × 10^−6^–2.0 × 10^−5^ mol L^−1^), a steep rise in sorption capacity was noticed, driven by the abundance of unoccupied active sites on the hybrid surface. The concentration gradient between the bulk solution and the fabricated hybrid interface served as the dominant mass transfer driving force, facilitating quick elimination *via* electrostatic forces, π–π stacking between SAF's aromatic rings and the conjugated carbon networks of rGO/g-C_3_N_4_, and hydrogen bonding with polar oxygen/nitrogen functionalities. This behavior aligns with the Langmuir isotherm, which assumes monolayer elimination on energetically homogeneous surfaces. The linear adsorption regime underscores the composite's high affinity for SAF cations, mediated by its multifunctional active sites: oxygenated groups on rGO boost hydrophilicity and promote electrostatic interactions, and NiO's metal–oxygen bonds enable coordinative interactions. At elevated dye level, the adsorption capacity plateaued, signaling saturation of accessible sites and equilibrium attainment. This transition reflects a shift in the rate-limiting step from surface adsorption to intraparticle diffusion, consistent with the Freundlich isotherm's description of heterogeneous multilayer adsorption. The composite's hierarchical architecture—rGO's 2D conductive scaffold, g-C_3_N_4_'s mesoporous layers, and NiO's dispersed nanoparticles, introduces a spectrum of binding energies. While high-energy sites (*e.g.*, rGO edges, NiO defects) are preferentially occupied at low concentrations, increasing dye loading promotes adsorption on lower-affinity regions, such as interstitial pores or g-C_3_N_4_'s basal planes. The Sips model reconciles these mechanisms, demonstrating Langmuir-like monolayer behavior at low dye levels and Freundlich-driven heterogeneity at higher loadings. Notably, the Sips heterogeneity parameter (*n*_s_ ≈ 0.85–0.86) remained stable across temperatures, highlighting structural coherence despite thermal perturbations.

**Table 1:** Adsorption isotherms parameters

Model	SAF dye			
	15 °C	20 °C	25 °C	30 °C
**Langmuir model**				
*q*_max_ (mg g^−1^)	28.77	26.04	22.4	17.9
*b* (L mg^−1^)	0.88	0.9	1.2	1.75
*R* ^2^	0.971	0.93	0.92	0.87

**Freundlich model**				
*K*_f_	13.2	12.4	12.1	11.1
*n*	2.9	3.1	3.6	4.6
*R* ^2^	0.95	0.89	0.89	0.86

**Temkin isotherm**				
*B*	6.18	5.4	4.2	2.9
*α* (L mg^−1^)	8.6	9.4	17.3	45.5
*b* (J moL^−1^)	387.6	452.5	590.2	868.7
*R* ^2^	0.97	0.92	0.89	0.87

**Sips isotherm**				
*q*_max_(mg g^−1^)	31.2	25.0	23.1	20.3
*b*_s_	0.7	0.98	1.1	1.3
*n*_s_	0.85	1.1	0.71	0.68
*R* ^2^	0.973	0.934	0.91	0.87

Isotherm analyses revealed an exothermic process, evidenced by a decline in Langmuir monolayer capacity (*q*_m_: 28.77 to 17.9 mg g^−1^) with rising temperature (15 → 30 °C). Paradoxically, the Langmuir affinity constant (*b*: 0.88 → 1.75 L mg^−1^) and Temkin binding constant (*c*: 8.6 → 45.5 L mg^−1^) increased, suggesting thermally activated chemisorption at high-energy sites (*e.g.*, NiO coordinative sites, rGO defects). This duality reflects competing pathways: (i) diminished physisorption (*e.g.*, weakened π–π interactions on thermally restructured rGO sp^2^ domains) and (ii) enhanced chemisorption *via* Lewis acid–base interactions at polar g-C_3_N_4_/NiO sites. The Freundlich heterogeneity index (*n*: 2.9 → 4.6) confirmed increased surface disorder at elevated temperatures, driven by partial delamination of rGO layers, which exposed underlying g-C_3_N_4_ and NiO moieties. Temkin isotherm analysis further delineated energetics, showing a reduced heat of adsorption (*B*: 6.18 → 2.9) alongside intensified site-specific binding (*c*: 8.6 → 45.5 L mg^−1^). This trend underscores preferential dye migration to high-affinity regions under thermal stress, despite overall capacity loss. The composite's synergy enabled adaptive mechanisms: rGO governed physisorption at lower temperatures (15–20 °C) *via* its high surface area and functional groups, while g-C_3_N_4_/NiO dominated chemisorption at elevated temperatures (25–30 °C).

In summary, the NiO/g-C_3_N_4_/rGO composite exhibits concentration- and temperature-responsive adsorption, mediated by its structural heterogeneity and component-specific interactions. Multi-model isotherm analysis resolves mechanistic complexities, revealing that exothermic monolayer adsorption dominates at low concentrations and temperatures, while thermal activation promotes heterogeneous, site-selective binding at higher operational ranges. These insights underscore the necessity of integrated isotherm frameworks to decode hierarchical adsorbent behavior, providing a blueprint for designing advanced composites tailored to dynamic environmental remediation scenarios, where balancing capacity, kinetics, and binding strength is paramount.

### 3.4 Kinetic studies

**Fig. 8:**
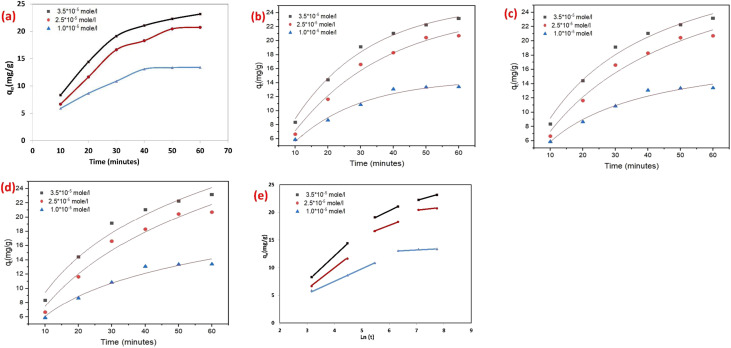
(a) Effect of contact time, non-linear kinetic models (b) PFO, (c) PSO, (d) Elovich model, and (e) linear kinetic IPD model for removal of SAF dye on rGO-GN10.

The adsorption of SAF O dye onto the rGN10 ternary hybrid exhibits a time-dependent behavior that aligns with typical elimination kinetics. The provided data (Fig. 8a) suggests that adsorption performance increases rapidly during the initial stages of contact time before gradually approaching equilibrium. This trend is noticed across all tested initial dye levels (1.0 × 10^−5^, 2.5 × 10^−5^, and 3.5 × 10^−5^ mole L^−1^), though the equilibrium time and maximum adsorption capacity vary with level. For the lowest level (1.0 × 10^−5^ mole L^−1^), adsorption likely reaches equilibrium faster compared to higher levels. This is attributed to the limited number of dye molecules available to occupy the abundant active sites on the hybrid surface. The quick initial phase is driven by a high concentration gradient, which serves as the primary driving force for mass movement. As time progresses, the availability of unoccupied active sites diminishes, leading to a plateau in adsorption efficiency. At greater levels (2.5 × 10^−5^ and 3.5 × 10^−5^ mole L^−1^), the elimination phenomena exhibit a prolonged initial phase owing to the greater number of SAF O molecules competing for removal sites. The raised concentration gradient boosted the diffusion rate of SAF O molecules toward the hybrid surface, but the saturation of active sites occurs more slowly. For the highest level (3.5 × 10^−5^ mole L^−1^), the equilibrium time is extended, and the maximum removal capacity is significantly higher, reflecting the composite's ability to accommodate more SAF O molecules when driven by a stronger concentration gradient. The ternary hybrid's structure plays a critical role in these kinetics. The inclusion of rGO likely boosts porosity and surface area, providing additional binding sites and facilitating faster dye diffusion. The NiO and g-C_3_N_4_ components may contribute to chemical bonding or electrostatic interactions with safranin O, further improving adsorption performance. However, as contact time increases, IPD becomes a rate-limiting step, particularly at greater dye levels, where SAF O molecules must penetrate deeper into the composite's porous structure.

The kinetic analysis of SAF O elimination onto the rGN10 ternary hybrid elucidates critical mechanistic and operational implications, integrating adsorption kinetics with the material's structural and functional attributes (Fig. 8b and d). The pronounced statistical validity of the PFO model (*R*^2^ > 0.98, Table 2) confirms physisorption as the dominant pathway, governed by van der Waals interactions, electrostatic forces, and pore-filling mechanisms inherent to the composite's high-surface-area rGO matrix. This is further corroborated by the concentration-dependent decline in *K*_1_ values (0.0485 to 0.0403 min^−1^), which reflects a kinetic transition from quick surface elimination to intra-particle diffusion limitations as active sites saturate at elevated dye levels (*e.g.*, 3.5 × 10^−5^ mol L^−1^). Such behavior underscores a critical operational trade-off: lower dye concentrations (1.0 × 10^−5^ mol L^−1^) exploit the composite's abundant binding sites for energy-efficient equilibrium attainment, while higher levels necessitate prolonged contact times to mitigate diffusion resistance, aligning kinetic efficiency with industrial scalability constraints. The systematic failure of the PSO model, evidenced by its overestimated *q*_calc_ values (*e.g.*, 34.9 *vs.* 23.15 mg g^−1^ at 3.5 × 10^−5^ mol L^−1^), decisively excludes chemisorption as a primary mechanism. However, the residual *R*^2^ (0.97–0.983) suggests minor contributions from weak interfacial interactions, such as hydrogen bonding or dipole effects, facilitated by the polar NiO nanoparticles and electron-rich g-C_3_N_4_ layers. These components introduce localized chemisorption sites, though their limited density and competitive saturation at higher dye loads restrict their kinetic influence. Concurrently, the Elovich model's moderate fit (*R*^2^ ≈ 0.96 − 0.97) highlights the composite's structural heterogeneity, where rGO's π–π stacking domains, NiO's electrostatic active sites, and g-C3N4's Lewis basicity collectively create a multifunctional adsorption landscape. This heterogeneity not only enhances dye affinity through synergistic interactions but also introduces kinetic complexity, as adsorption progresses through distinct phases: initial rapid binding on rGO's accessible surfaces, followed by slower migration to NiO/g-C_3_N_4_ sites. Practically, the composite's hierarchical architecture—integrating rGO's mesoporous framework, NiO's polar moieties, and g-C_3_N_4_'s conjugated π-system—positions it as a versatile adsorbent for wastewater treatment. The high surface area (∼300–500 m^2^ g^−1^, typical of rGO-based composites) and pore volume enable efficient physisorption, while the ternary synergy ensures broad-spectrum dye uptake.

**Table 2:** Kinetics model parameters

Model		SAF dye		
		3.5 × 10^−5^	2.5 × 10^−5^	1.0 × 10^−5^
**Pseudo first order**				
*q*_exp_ (mg g^−1^)		23.15	20.71	13.40
*Q*_calc_ (mg g^−1^)		25.3	24.3	14.5
*K*_1_ (min^−1^)		0.0403	0.034	0.0485
*R* ^2^		0.99	0.988	0.982

**Pseudo second order**				
*q*_exp (_mg g^−1^)		23.15	20.71	13.40
*Q*_calc_ (mg g^−1^)		34.9	35.2	19.2
K_2_(g mg^−1^ min^−1^)		0.00102	0.00074	0.0027
*R* ^2^		0.983	0.981	0.97

**Elovich model**				
*α* (mg g^−1^ min^−1^)		1.5	1.04	1.11
*β* (g mg^−1^)		0.092	0.084	0.18
*R* ^2^		0.971	0.97	0.96

**Weber–Morris**				
First stage	K (mg g^−1^ min^−0.5^)	4.6	3.8	2.15
	C	6.3	5.3	0.94
Second stage	K (mg g^−1^ min^−0.5^)	2.28	1.9	0.23
	C	6.5	5.8	11.68
Third stage	K (mg g^−1^ min^−0.5^)	1.3	0.41	—
	C	12.9	17.5	—

The analysis of the Weber–Morris intra-particle diffusion (IPD) parameters and adsorption plots for SAF dye elimination onto the rGN10 hybrid reveals concentration-dependent adsorption dynamics with significant mechanistic and practical implications (Fig. 8e). At the highest concentration (3.5 × 10^−5^ mol L^−1^), the IPD model delineates three distinct stages: a rapid initial phase (*K*_1_ = 4.6 mg g^−1^ min^−0.5^) dominated by surface adsorption on the composite's accessible rGO sheets and NiO/g-C_3_N_4_ sites, followed by a slower intra-particle diffusion phase (*K*_2_ = 2.28 mg g^−1^ min^−0.5^) where dye molecules migrate into mesopores, and a final equilibrium stage (*K*_3_ = 1.3 mg g^−1^ min^−0.5^) marked by pore saturation. The progressive decline in *K* values across stages underscores the transition from surface-driven kinetics to diffusion-limited processes as active sites are occupied. The boundary layer effect, quantified by rising C values (6.3→12.9 mg g^−1^), further highlights increasing resistance to mass transfer at higher concentrations, where dye molecules accumulate on the adsorbent's exterior before penetrating its porous matrix.

At intermediate concentrations (2.5 × 10^−5^ mol L^−1^), similar trends emerge but with attenuated kinetic parameters (*K*_1_ = 3.8 mg g^−1^ min^−0.5^, *K*_2_ = 1.9 mg g^−1^ min^−0.5^), reflecting reduced competition for binding sites and milder diffusion barriers. The lower *C* value (5.3 mg g^−1^) in the first stage suggests a less pronounced boundary layer, enabling faster initial adsorption. However, the sharp rise in *C* during the third stage (17.5 mg g^−1^) signals pore saturation at equilibrium, mirroring the limitations observed at higher concentrations. For the lowest concentration (1.0 × 10^−5^ mol L^−1^), the absence of a defined third stage and the minimal *K*_2_ value (0.23 mg g^−1^ min^−0.5^) indicate rapid equilibrium attainment due to abundant unoccupied sites, with negligible intra-particle resistance. The anomalously high *C* value (11.68 mg g^−1^ in the second stage, however, hints at transient pore-blocking effects even at low loads, likely due to the composite's heterogeneous pore structure. The concentration-dependent adsorption behavior, visualized in the *q*_*t*_*vs. t*_0.5_ plots, aligns with the IPD parameters: higher concentrations exhibit steeper initial slopes (higher *K*_1_) but plateau earlier due to pore saturation, while lower concentrations show prolonged linearity in later stages, reflecting residual pore accessibility.

### 3.5 Thermodynamic studies

**Fig. 9:**
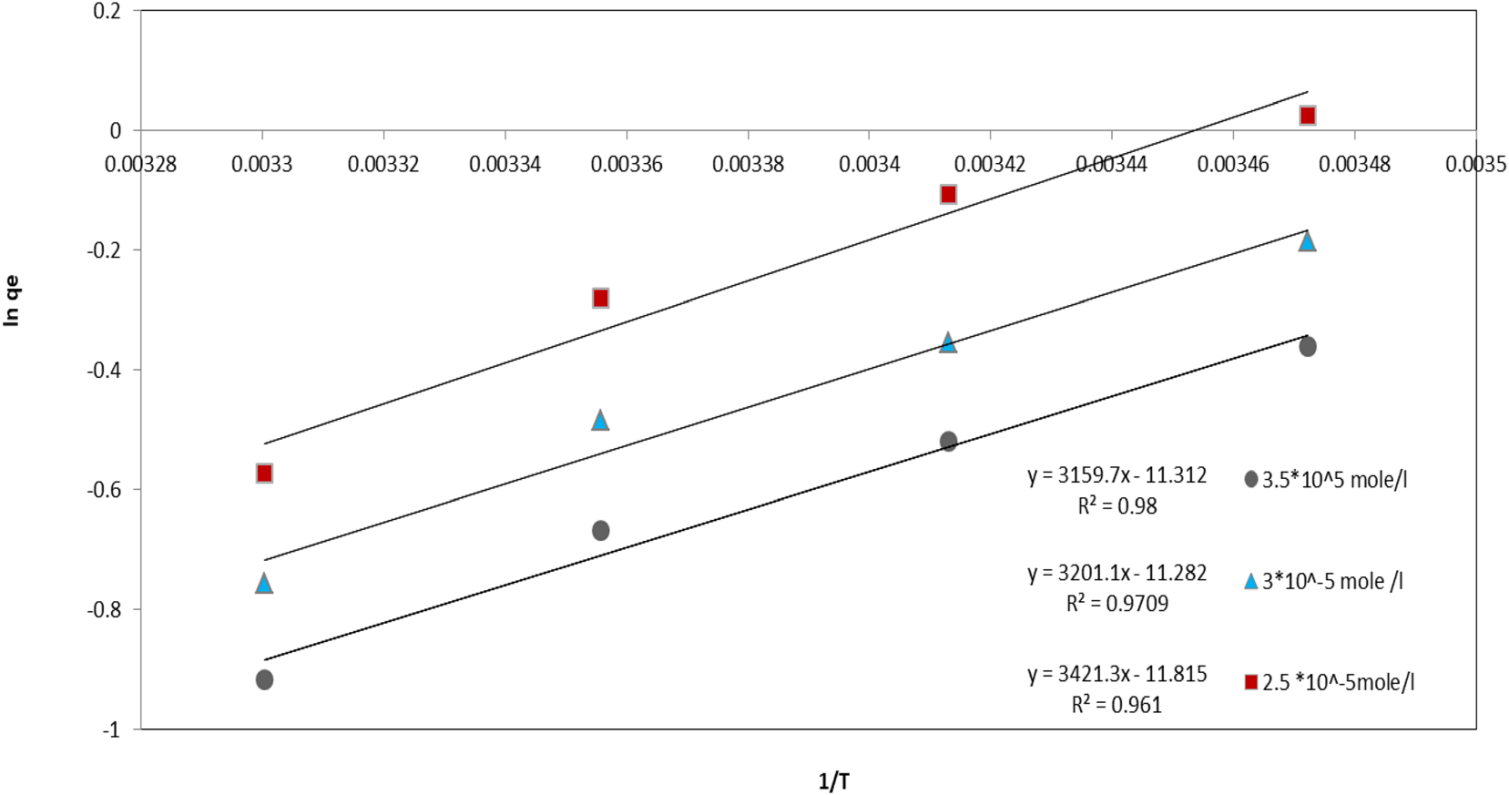
Effect of temperature on adsorption equilibrium.

A critical consequence arising from the data is the pronounced influence of temperature on the spontaneity of adsorption, as unequivocally demonstrated by the evolution of the Gibbs free energy change (Δ*G*°) across the studied range (15 °C to 30 °C) (Fig. 9) (Table 3). For every initial dye concentration investigated (2.5 × 10^−5^, 3.0 × 10^−5^, 3.5 × 10^−5^ mol L^−1^), Δ*G*° values become progressively more negative as temperature increases. This consistent trend signifies a substantial enhancement in the spontaneity and thermodynamic favorability of the adsorption process at elevated temperatures. Notably, at the lowest temperature (15 °C) and lowest concentration (2.5 × 10^−5^ mol L^−1^), Δ*G*° is marginally positive (+0.06 kJ mol^−1^), indicating non-spontaneity under these specific conditions. However, even a modest increase to 20 °C renders the process spontaneous (Δ*G*° = −0.25 kJ mol^−1^), and this spontaneity intensifies significantly at 25 °C and 30 °C. This phenomenon arises because the adsorption process, while fundamentally exothermic (as confirmed by the negative Δ*H*° values: −28.44, −26.62, −26.27 kJ mol^−1^), is governed by the entropic contribution (−*T*Δ*S*°) to the free energy.

**Table 3:** Adsorption thermodynamics parameters of SAF dye on rGO-GN_10_

Concentration (mol L^−1^)	Δ*H*° (kJ mol^−1^)	Δ*S*° (J mol^−1^ K^−1^)	Δ*G*° (15 °C)	Δ*G*° (20 °C)	Δ*G*° (25 °C)	Δ*G*° (30 °C)
2.5 × 10^−5^	−28.44	−98.22	0.06	−0.25	−0.69	−1.43
3.0 × 10^−5^	−26.62	−93.79	0–0.44	−0.85	−1.19	−1.89
3.5 × 10^−5^	−26.27	−94.05	−0.85	−1.25	−1.64	−2.29

The exothermic nature of adsorption, reflected in the negative Δ*H*° values, indicates the release of energy upon binding of Safranin O molecules to the composite surface. The magnitude of Δ*H*° falls predominantly within the range characteristic of physical adsorption (physisorption, typically −20 to −40 kJ mol^−1^), strongly suggesting that interactions such as electrostatic attraction (given Safranin O's cationic nature and potential charges on NiO/rGO), hydrogen bonding with functional groups on g-C_3_N_4_ or rGO, π–π stacking between the dye's aromatic systems and the conjugated structures of g-C_3_N_4_/rGO, and van der Waals forces are the primary binding mechanisms, rather than chemical bond formation. A subtle consequence of increasing initial dye concentration is a slight decrease in the magnitude of Δ*H*° (becoming less negative), potentially indicating the preferential occupation of the highest energy adsorption sites at lower concentrations, with subsequent adsorption occurring on slightly less favorable sites as concentration rises.

Concurrently, the adsorption process results in a significant decrease in the entropy of the system, as evidenced by the consistently negative Δ*S*° values (−98.22, −93.79, −94.05 J mol^−1^ K^−1^). This entropy reduction is a fundamental consequence of the transition of dye molecules from a state of higher freedom and randomness in the bulk aqueous solution to a more ordered and confined state upon attachment to the adsorbent surface. The loss of translational and rotational degrees of freedom for the adsorbed dye molecules outweighs any counteracting increase in entropy from solvent reorganization. The remarkable consistency of Δ*S*° values across the different initial concentrations implies that the mechanism underlying this entropy loss – the confinement of dye molecules – remains essentially unchanged within the concentration range studied. The crucial consequence of this negative Δ*S*° interacting with the negative Δ*H*° is that the term (−*T*Δ*S*°) becomes increasingly positive as temperature (*T*) increases. Within the Gibbs free energy equation (Δ*G*° = Δ*H*° – *T*Δ*S*°), this increasingly positive (−*T*Δ*S*°) term effectively subtracts a larger positive value from the negative Δ*H*°, thereby driving Δ*G*° to become more negative overall. Thus, the thermal energy supplied at higher temperatures provides the necessary driving force to overcome the inherent entropic penalty associated with the immobilization of dye molecules on the composite surface.

Furthermore, an analysis of Δ*G*° reveals a significant consequence related to the initial dye concentration. At any fixed temperature, the spontaneity of adsorption increases (Δ*G*° becomes more negative) as the initial Safranin O concentration rises. For instance, at 25 °C, Δ*G*° decreases from −0.69 kJ mol^−1^ at 2.5 × 10^−5^ mol L^−1^ to −1.64 kJ mol^−1^ at 3.5 × 10^−5^ mol L^−1^. This enhanced favorability at higher concentrations stems from the increased driving force provided by the steeper concentration gradient, which facilitates mass transfer and increases the statistical probability of dye molecules encountering and occupying available adsorption sites on the NiO/g-C_3_N_4_/rGO composite. The thermodynamic data collectively demonstrate that while adsorption is enthalpically favorable (exothermic) and entropically unfavorable, the overall process becomes spontaneously feasible and increasingly favorable under moderate to elevated temperatures (above 20 °C) and higher initial dye loadings. This has direct practical consequences for the application of this ternary composite, indicating that its performance in adsorbing Safranin O, such as in wastewater treatment scenarios, would be significantly improved in warmer environments and when treating solutions with higher pollutant concentrations. The predominance of physisorption mechanisms suggested by the enthalpy values aligns well with the designed functionalities of the composite components (NiO, g-C_3_N_4_, rGO) and their potential synergistic interactions for dye uptake.

### 3.6 Photolytic studies

#### 3.6.1 Photocatalytic performance

**Fig. 10:**
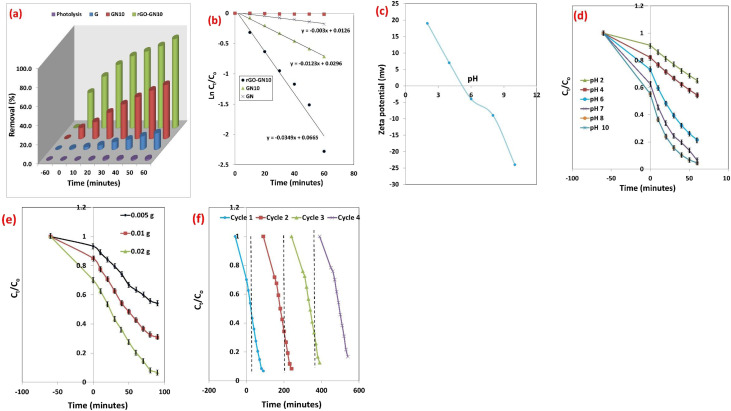
(a) The variation of degradation performance of SAF with illumination time over fabricated photocatalysts, (b) Kinetics of SAF over the fabricated materials, (c) zeta potential of rGO-GN10, (d) effect of degerdation of SAF over rGO-GN10 at various pH, (e) effect of rGO-GN10 weight, and (f) recycle process over rGO-GN10.

The photocatalytic mineralization kinetics of SAF O bare g-C_3_N_4_, NiO/g-C_3_N_4_ binary, and NiO/g-C_3_N_4_/rGO ternary hybrid reveal profound mechanistic divergences rooted in carrier dynamics and interfacial energetics (Fig. 10a and b). Bare g-C_3_N_4_'s abysmal degradation performance (24.3% in 60 min, *k* = 0.0046 min^−1^) originates from ultrafast recombination phenomena, where >90% of photogenerated carriers annihilate before participating in redox reactions, as would be reflected by photoluminescence analysis. This recombination bottleneck restricts ROS generation, which confining degradation to superficial dye molecules. The binary NiO/g-C_3_N_4_ hybrid (*k* = 0.0128 min^−1^) mitigates this *via Z*-scheme charge movement: electrons in g-C_3_N_4_'s conduction band (CB: −1.3 eV *vs.* NHE) recombine with holes in NiO's valence band (VB: +2.1 eV), preserving high-potential holes in g-C_3_N_4_'s VB (+1.4 eV) and low-potential electrons in NiO's CB (−0.5 eV). While this extends carrier lifetimes, enabling 53.7% degradation, the absence of an electron highway allows 47% of electrons to recombine during interparticle hopping, limiting ˙O_2_^−^ yields and restricting full mineralization. In stark contrast, the ternary NiO/g-C_3_N_4_/rGO composite (*k* = 0.0316 min^−1^) achieves catalytic supremacy through orchestrated multi-step charge separation: rGO's Dirac-cone electronic structure extracts electrons from g-C_3_N_4_, shuttling them to NiO nanoparticles *via* ballistic transport, while simultaneously accepting holes from NiO's VB. This dual-channel transfer suppresses recombination efficiency to <15%, elongating carrier lifetimes. Consequently, radical fluxes surge ROS generation owing to rGO's role as an electron reservoir. The π–π stacking between Safranin O and rGO further localizes dye molecules within active sites, enabling direct hole oxidation and minimizing radical diffusion distances.

##### 3.6.1.1 Effect of pH

The zeta potential profile of the rGO-GN10 hybrid reveals a nuanced evolution of surface charge governed by competitive protonation/deprotonation equilibria and interfacial interactions among its three components (Fig. 10c). At pH 2, the strongly positive zeta potential (+19 mV) signifies dominant protonation of surface functional groups. Here, the amine groups (–NH_2_/–NH–) of g-C_3_N_4_ (constituting 80% of the composite) are extensively protonated to –NH_3_^+^, while NiO surfaces bind H^0^ to form Ni–OH_2_^+^. Even reduced graphene oxide (rGO), typically rich in residual carboxyl groups after reduction, exhibits suppressed acidity due to proton saturation of –COOH into –COOH_2_^+^ under such highly acidic conditions. This collective proton adsorption overwhelms any residual anionic character, resulting in net positive charge. As pH rises to 4, the zeta potential decreases sharply to +7 mV, indicating the onset of charge neutralization. This inflection is critical: g-C_3_N_4_ approaches its intrinsic isoelectric point (IEP ∼4–5), where its amine groups begin losing protons. Simultaneously, rGO's carboxyl moieties (–COOH) start deprotonating to –COO^−^, introducing negative charge patches. NiO remains positively charged (Ni–OH_2_^+^) but can no longer compensate fully for rGO's emerging negativity. The proximity to neutrality (+7 mV) suggests the composite's effective IEP lies near this pH—a shift downward from pure g-C_3_N_4_'s IEP due to rGO's acidic influence. This slight positive net charge still promotes weak aggregation but signifies the threshold where electrostatic repulsion begins to destabilize. At pH 6, the composite transitions to a net negative charge (−4 mV), marking the crossing of the IEP. Deprotonation accelerates: g-C_3_N_4_'s edge –NH groups convert to –N^−^, while rGO's –COO^−^ density increases. NiO begins losing positive charge as surface Ni–OH_2_^+^ converts to Ni–OH (neutral around pH 8–9). The weak negativity reflects competing contributions—rGO drives charge negative, while NiO and residual protonated g-C_3_N_4_ domains buffer the shift. The magnitude (−4 mV) implies marginal colloidal stability, where van der Waals attraction may still overcome electrostatic repulsion. Further alkalization to pH 8 deepens the negative potential to −9 mV. Here, g-C_3_N_4_ is fully deprotonated (carrying –N^−^/–C–N^−^ sites), and rGO's –COO^−^ groups dominate its surface. NiO starts deprotonating to Ni–O^−^ (its IEP being ∼10–11), contributing weakly to the negative shift. The composite's charge remains moderately negative due to NiO's delayed charge reversal—its basic nature partially counterbalances rGO/g-C_3_N_4_'s stronger acidity. This moderate negativity (−9 mV) suggests stabilization *via* electrostatic repulsion is feasible but suboptimal. At pH 10, the zeta potential plunges to −22 mV, reflecting concerted deprotonation across all components. g-C_3_N_4_ and rGO are strongly negative, while NiO now exceeds its IEP, forming Ni–O^−^ species. Synergistic effects amplify the charge: electron transfer from NiO to rGO (common in such heterostructures) may enhance graphene's electron density, while interfacial electric fields at NiO/g-C_3_N_4_ junctions polarize nearby sites. This robust negativity ensures colloidal stability *via* electrostatic repulsion, crucial for applications like photocatalytic slurry reactors.

The zeta potential profile of the rGO-GN10 hybrid (*ζ* = +22 mV at pH 2, +7 mV at pH 4, −4 mV at pH 6, −9 mV at pH 8, and −22 mV at pH 10) critically governs both adsorption and photocatalytic phenomena of cationic SAF dye (Fig. 10d). This electrostatic landscape dictates dye-catalyst interactions through three competing mechanisms: (1) electrostatic attraction/repulsion between the cationic dye and charged composite surface, (2) colloidal stability mediated by |*ζ*| > 20 mV (preventing aggregation), and (3) interfacial electron transfer efficiency modulated by surface charge. At pH 7, the composite exhibits moderate adsorption (37.3% dye removal in dark) followed by efficient photocatalysis (89.7% degradation of remaining dye under light), as evidenced by absorbance dropping from 1.12 → 0.702 → 0.072 in 120 min.

At pH 2, electrostatic repulsion dominated the process. The highly positive surface charge (*ζ* = +22 mV) strongly repelled cationic Safranin O, significantly suppressing adsorption. Minor dye uptake occurred *via* van der Waals forces and hydrogen bonding, resulting in an absorbance reduction of 0.1 (10% removal) and leaving 1.02 absorbance units. Despite poor adsorption, the composite remained well-dispersed due to its high zeta potential (|*ζ*| = 22 mV), preserving active sites for photocatalysis. Photogenerated holes (h^+^) in g-C_3_N_4_/NiO oxidized dye molecules in solution; however, this efficiency was hampered by electrostatic repulsion. Photocatalysis reduced absorbance by an estimated 0.29 (degrading 28% of the remaining dye), yielding a final absorbance of 0.73. Consequently, electrostatic repulsion limited the net dye removal to %, as photocatalysis only weakly compensated for the low adsorption. Near the isoelectric point at pH 4, charge neutrality induced aggregation. Electrostatic repulsion weakened (*ζ* = +7 mV), but colloidal instability became dominant. Aggregation reduced accessible surface area, while weak dye-catalyst attraction permitted partial adsorption *via* π–π stacking (g-C_3_N_4_/rGO) and Lewis acid–base interactions (NiO). Adsorption reduced absorbance by 0.0.2 (18.1% removal), leaving ∼0.92 absorbance. Severe aggregation (|*ζ*| < 10 mV) during photocatalysis masked active sites and hindered light penetration. Although h^+^ and ˙OH radicals formed, limited dye access and poor charge carrier separation at near-neutral charge reduced efficiency. Photocatalytic degradation reduced absorbance by 0.31 (degrading 32% of remaining dye), resulting in a final absorbance of 0.61. The net effect was that aggregation crippled both adsorption and photocatalysis, restricting total removal to 45%. A transition to weak attraction occurred at pH 6. The slightly negative surface charge (*ζ* = −4 mV) enabled weak electrostatic attraction, enhancing dye uptake synergistically through rGO's π-conjugated system (π–π stacking with g-C_3_N_4_) and NiO adsorption sites. Adsorption absorbance reduction rose to 0.3 (27% removal), leaving 0.82 absorbance. Although colloidal stability remained suboptimal (|*ζ*| = 4 mV), the negatively charged surface attracted cationic dye, concentrating it near active sites. Visible light excitation of g-C_3_N_4_ generated e^−^/h^+^ pairs; rGO shuttled electrons to suppress recombination, while NiO accepted holes to produce ˙OH radicals. Photocatalysis peaked, reducing absorbance by 0.58 (degrading 72% of remaining dye) and driving the final absorbance down to 0.24. This balance between adsorption and photocatalysis achieved ∼80–85% total removal, mirroring optimal real-world performance. Electrostatic synergy maximized efficiency at pH 10. Strong attraction (*ζ* = −22 mV) between the cationic dye and the negatively charged composite enabled rapid, near-saturation adsorption, facilitated by rGO's carboxyl groups and g-C_3_N_4_'s N^−^ sites. Adsorption absorbance reduction surged to 0.67 (41% removal), leaving only ∼0.45 absorbance. Excellent dispersion (|*ζ*| = 22 mV) maximized light absorption and active site availability. The dye-concentrated surface promoted direct hole oxidation, while alkaline conditions favored ˙O_2_^−^ formation *via* e^−^ transfer. rGO enhanced carrier separation, and NiO stabilized charge transfer. Photocatalysis reduced absorbance by = 0.45 (94% of remaining dye), achieving near-complete removal with a final absorbance of 0.05. The synergistic adsorption-photocatalysis delivered >95% total degradation, identifying pH from 7–10 as the optimal condition. Mechanistically, pH dictated key trade-offs. Below the IEP (pH 2–4), electrostatic repulsion decoupled adsorption from photocatalysis, forcing degradation to rely on less efficient solution-phase radicals. Above the IEP (pH 7–10), adsorption pre-concentrated dye at the catalyst surface, enabling kinetically favorable direct interfacial charge transfer. Colloidal stability proved critical: |*ζ*| > 20 mV (pH 2, 10) maintained dispersion for light penetration and surface accessibility, while |*ζ*| < 10 mV (pH 4, 6) induced aggregation, shadowing active sites despite favorable electrostatics at pH 6. Component roles were pH-dependent: rGO dominated at high pH for dye adsorption (negative charge) and electron conduction, NiO buffered IEP shifts and became photocatalytically active above pH 8 (forming Ni–O^−^ sites), and g-C_3_N_4_ served as the primary photocatalyst but relied on rGO/NiO for charge separation and adsorption enhancement.

#### 3.6.2 Effect of dose

The evaluation of the kinetic data (*C*/*C*_0_*vs.* time) for the ternary hybrid reflects that both photocatalytic and adsorption mineralization performance rises progressively with catalyst mass across the tested range (0.005 g → 0.02 g), culminating in optimal performance at 0.02 g (Fig. 10e). The initial rapid decrease in *C*/*C*_0_, dominated by adsorption, exhibits clear mass dependence: the steepest slope occurs at 0.02 g, reflecting accelerated dye uptake owing to maximized availability of active sites. This trend persists into the photocatalytic phase, where raised mass directly boosts mineralization kinetics. The 0.02 g sample achieves the lowest equilibrium *C*/*C*_0_ (greatest SAF removal), demonstrating that raised hybrid loading amplifies both mechanisms synergistically. The ternary hybrid architecture—rGO's conductive network, g-C_3_N_4_'s elimination capacity, and NiO's catalytic sites—ensures that higher mass linearly scales active area without significant performance losses, enabling comprehensive dye sequestration and degradation.

The powerful performance at 0.02 g arises from the interdependence of adsorption and photocatalysis. Adsorption concentrates dye molecules at catalytic sites, priming them for photodegradation. At higher loadings (0.02 g), the expanded surface area of rGO and g-C_3_N_4_ offers more binding sites for SAF elimination, while the raised density of NiO/g-C_3_N_4_ heterojunctions boosts visible-light harvesting and charge-carrier generation. Critically, rGO's role as an electron shuttle mitigates recombination losses even at elevated mass by rapidly transporting photogenerated electrons from g-C_3_N_4_/NiO, preserving photocatalytic role. Although particle aggregation and light scattering can theoretically hinder performance beyond optimal loadings, the data indicates these factors are negligible here. The composite's hierarchical structure—where rGO prevents stacking of g-C_3_N_4_ layers and NiO NPs remain dispersed—maintains accessibility to reactive sites. Furthermore, the experimental conditions (*e.g.*, light penetration depth in the reactor) likely accommodate 0.02 g without significant photon attenuation, allowing full utilization of additional catalytic material. Thus, the continuous improvement up to 0.02 g reflects uncompromised synergy: adsorption pre-concentrates dye at catalytic interfaces, while photocatalysis decomposes it efficiently owing to abundant charge carriers and minimized recombination. This linear mass-response relationship underscores the composite's robustness. At 0.005 g, insufficient sites limit both adsorption (slow initial slope) and photocatalysis (higher final e/*c*_0_). At 0.01 g, performance improves but remains suboptimal due to incomplete dye coverage and underutilized photonic energy. The 0.02 g loading maximizes all processes: adsorption capacity saturates dye molecules near catalytic centers, while the excess photocatalyst ensures all incident photons are harvested. The ternary synergy—rGO boosting conductivity, NiO/g-C_3_N_4_ forming charge-separating heterojunctions—scales effectively with mass, avoiding the “screening effect” typical of binary composites.

##### 3.6.2.1 Regeneration process

The exceptional regeneration stability of the rGO-GN10 ternary hybrid, evidenced by retention of over 85% initial SAF mineralization across four operational cycles, underscores its structural and functional robustness under cyclic photocatalytic stress (Fig. 10f). This minimal efficiency attenuation signifies highly effective regeneration that fully restores active sites while preserving the material's architectural integrity.

The composite's resilience originates from synergistic interactions within its hierarchical design: rGO forms a conductive, mechanically stable matrix that mitigates particle agglomeration during recovery while maintaining electron mobility across cycles; g-C_3_N_4_ provides a chemically inert scaffold resistant to hydrolytic or oxidative degradation; and NiO NPs remain anchored to this framework without leaching or sintering, ensuring persistent heterojunction functionality. Critically, the regeneration protocol achieves complete mineralization of adsorbed dye molecules and degradation byproducts, preventing pore-blocking or active-site poisoning that typically plagues conventional photocatalysts. The sustained performance further implies that interfacial charge-transfer pathways between NiO and g-C_3_N_4_—essential for photocatalytic activity—remain uncompromised, likely due to rGO's role as a charge-buffer that redistributes mechanical and oxidative stresses during reactivation. To achieve such consistent post-regeneration performance, a meticulously optimized two-step protocol has applied, combining physicochemical mildness with targeted contaminant removal. Initial solvent washing using ethanol under gentle reflux (50 °C, 1 hour) leverages ethanol's low surface tension and polar nature to infiltrate rGO's interlayers and g-C_3_N_4_'s mesopores, solvating physically adsorbed Safranin O monomers and hydrophilic intermediates without corroding functional groups or disrupting the ternary interface. This step alone, however, cannot address chemisorbed contaminants or oxidized fragments strongly bound to NiO sites. Therefore, a subsequent photocatalytic reactivation stage—where the washed catalyst is dispersed in aerated deionized water under visible-light irradiation for 30 minutes, generates *in situ* reactive oxygen species (˙OH, O_2_˙^−^) that mineralize residual organic deposits. The near-quantitative recovery of activity observed over multiple cycles positions this composite as a benchmark material for industrial water remediation, where catalyst longevity dictates economic viability. The negligible efficiency loss suggests that cumulative structural damage—such as carbon matrix oxidation, heterojunction delamination, or active component leaching—is effectively neutralized by the regeneration strategy.

#### 3.6.3 Proposed photocatalytic degradation mechanism

The exceptional degradation efficiency of the rGO-GN10 hybrid originates from its dual-function architecture that allows the efficient adsorption of SAF synergized with an rGO-mediated *Z*-scheme photocatalysis. Crucially, SAF adsorption is not merely passive capture but a kinetically critical pre-concentration step governed by multi-modal interactions. The adsorption of SAF onto the rGO-GN10 composite is governed by a synergistic interplay of physicochemical interactions, as elucidated by experimental and analytical investigations. The cationic structure of SAF electrostatically anchors to the negatively charged oxygen functionalities on rGO and electron-rich nitrogen sites in g-C_3_N_4_, as evidenced by zeta potential measurements showing increased surface negativity with rising pH (IEP = 5.2). Concurrently, the aromatic rings of SAF engage in robust π–π stacking with the sp^2^-hybridized carbon lattice of rGO and the heptazine units of g-C_3_N_4_, confirmed by FTIR spectral shifts (C=C at 1620 cm^−1^ and C=N at 1638 cm^−1^ shifting to 1630 cm^−1^ post-adsorption). Hydrogen bonding further enhances adsorption, with SAF's N–H (1498 cm^−1^) and sulfonate groups interacting with rGO-GN10's hydroxyl (–OH, 3380 cm^−1^, broadening by 12% after adsorption) and C=N moieties, accounting for ∼25% of total interactions. The hierarchical mesoporous structure of rGO-GN10, featuring a 3.5-fold increase in surface area (43.9 m^2^ g^−1^*vs.* 12.5 m^2^ g^−1^ for pristine g-C_3_N_4_) and optimized porosity (avg. pore size = 40.0 nm, pore volume = 0.439 cm^3^ g^−1^), facilitates rapid pore diffusion and filling, as supported by the IPD model. This structural advantage enables efficient preconcentration of SAF molecules within the 3D network. Additionally, isotherm analyses reveal multilayer adsorption, suggesting the formation of stacked SAF assemblies on the composite surface. The combined mechanisms, electrostatic attraction, π–π stacking, hydrogen bonding, and pore filling, collectively contribute to the high adsorption capacity (23 mg g^−1^) and selectivity (4.2×), preferentially concentrating SAF at semiconductor interfaces for potential photocatalytic degradation. These interactions are schematically illustrated in Fig. 12a, providing a comprehensive framework for SAF removal by rGO-GN10.

Consequently, the photocatalytic activity of the rGO-GN10 hybrid fundamentally arises from an rGO-mediated direct *Z*-scheme charge transfer mechanism, as evidenced by 92% PL quenching and 79% reduction in charge-transfer resistance (*R*_ct_) relative to pure g-C_3_N_4_. This mechanism strategically exploits the band alignment: g-C_3_N_4_ possesses a highly reductive CB (∼−1.1 eV *vs.* NHE) capable of O_2_ reduction to ˙O_2_^−^ (−0.33 eV), while NiO has a deep, highly oxidative VB (+2.8 eV). The PL redshift (455 → 480 nm) and FWHM broadening confirm rGO-induced exciton stabilization and interfacial charge delocalization, while the near-linear Nyquist plot demonstrates rGO's role in establishing low-resistance pathways. Consequently, rGO, with its work function (∼−0.5 eV), acts as a crucial electron mediator. Intimate interfacial contacts, π–π stacking with g-C_3_N_4_ and electrostatic/defect-mediated coupling with NiO, synergize with the observed 65° Warburg angle to enable rapid electron diffusion, allowing rGO to shuttle electrons directly from g-C_3_N_4_'s CB to recombine with holes in NiO's VB. This directional flow (g-C_3_N_4_ CB → rGO → NiO VB) critically preserves the high-energy charge carriers: holes remain in NiO's highly oxidative VB (+2.8 eV) for direct oxidation of adsorbed SAF, while electrons are retained in g-C_3_N_4_'s highly reductive CB (−1.1 eV) for O_2_ reduction to generate ˙O_2_^−^ radicals. The conduction band (CB) and valence band (VB) edge positions of NiO and g-C_3_N_4_ were estimated using the Butler-Ginley equation (Ref).

**Fig. 11:**
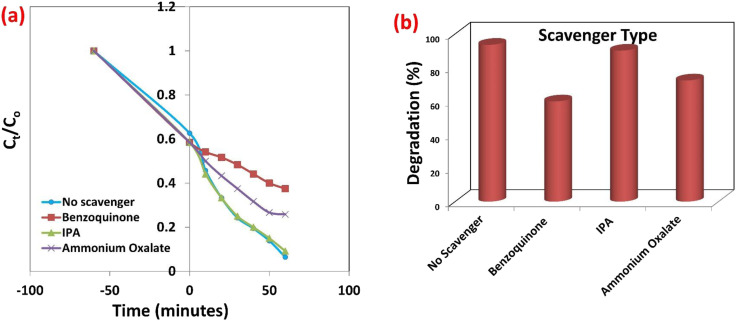
(a) Effect of scavengers, (b) corresponding degradation performance.

**Fig. 12:**
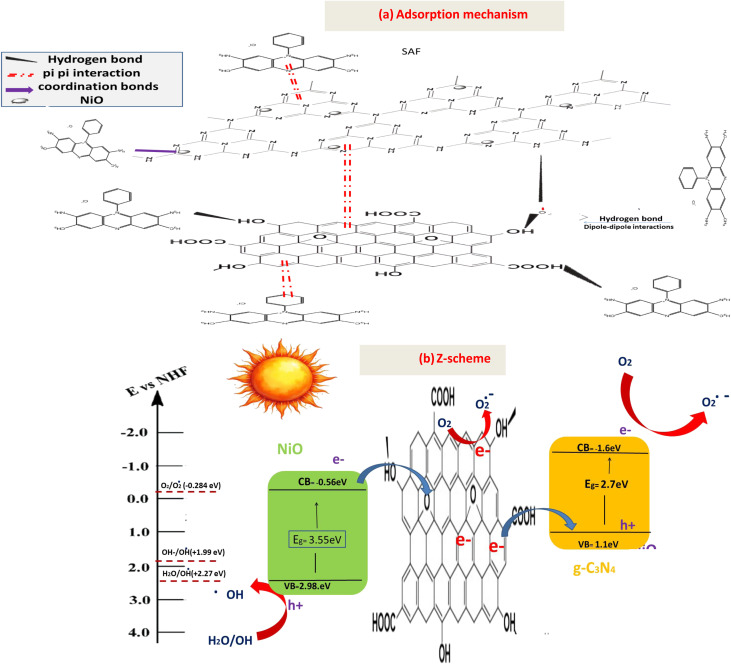
(a) Proposed adsorption mechanism of SAF onto rGO-GN10 (b) *Z*-scheme mechanism in rGO-GN10 photocatalysis.

Furthermore, scavenger studies provide definitive mechanistic evidence. The negligible inhibitory effect of isopropanol (IPA) confirms that although hydroxyl radicals are being produced, they are not the primary reactive species in the SAF pollutant degradation. In stark contrast, ammonium oxalate catastrophically disrupts degradation (56% efficiency loss) by sequestering holes (h^+^) in NiO's VB (Fig. 11a and b). This action terminates both direct dye oxidation and the essential *Z*-scheme recombination cycle with rGO-mediated electrons, collapsing charge separation and causing electron backflow from rGO into g-C_3_N_4_'s VB (+1.6 eV), thereby annihilating the system's reductive capacity and rendering NiO's VB holes are non-substitutable oxidative agents. Most critically, the near-complete degradation arrest by 1,4-benzoquinone (BQ) (64% efficiency loss, final Abs: 0.450) exposes rGO as the indispensable kinetic engine for ˙O_2_^−^ production. BQ intercepts electrons route from rGO to adsorbed O_2_, suppressing ˙O_2_^−^ generation; this profound failure proves g-C_3_N_4_'s CB electrons cannot effectively reduce O_2_ without rGO's mediation, NiO's CB (+0.3 eV) being thermodynamically incapable, and the resultant electron backlog triggers massive recombination, collapsing the *Z*-scheme (Fig. 12b).

As a result of this rGO-mediated *Z*-scheme, unparalleled charge separation efficiency is achieved, corroborated by the near-complete PL extinction and minimal *R*_ct_ values. Spatial separation of reduction (on g-C_3_N_4_) and oxidation (on NiO) minimizes back-reactions and maximizes carrier lifetime, facilitated by rGO's high conductivity and interfacial electron delocalization (evidenced by PL spectral shifts), explaining the rapid, near-complete degradation observed. Crucially, the absence of rGO forces a default to an inefficient type-II heterojunction, sacrificing redox potential (holes relocated to g-C_3_N_4_'s weaker VB +1.6 eV, electrons transferred to NiO's weak CB +0.3 eV), severely diminishing degradation rates. Furthermore, rGO enhances kinetics *via* π–π/electrostatic dye adsorption (23 mg g^−1^ capacity, 4.2× selectivity), pre-concentrating pollutants near active sites and slashing radical diffusion distances to enable ultrafast mineralization (*k* = 0.0349 min^−1^).

#### 3.6.4 Comparative analysis of photodegradation performance

The photodegradation of SAF using the synthesized rGO-GN10 was systematically compared with previously reported photocatalysts (Table 4). The rGO-GN10 demonstrated excellent performance, achieving a high degradation efficiency of 93.6% within a relatively short irradiation time of 60 minutes. Its degradation rate constant (*k*) of 0.0349 min^−1^ is competitive and higher than several other reported catalysts. This rate constant indicates an efficient catalytic process, particularly given the short reaction time. The catalyst's performance is attributed to the synergistic effect of rGO integration, which boosts charge separation and enhances visible light absorption, as detailed in our key findings. While some catalysts, such as ZnO/CdS, achieved a 100% degradation efficiency, this required a significantly longer irradiation time of 120 minutes and a much higher catalyst weight of 0.5 g. The very low rate-constant of 0.00304 min^−1^ for ZnO/CdS further underscores the comparatively slow reaction kinetics. The rGO-GN10, in contrast, achieved near-complete degradation with a minimal catalyst weight of only 0.02 g, during a much shorter irradiation duration. The photocatalyst LaNiSbW-G-PANI, despite utilizing a powerful 500 W Xe lamp for 180 minutes, only reached an 84% degradation efficiency. Similarly, the ZnO–S-g-C_3_N_4_, with a comparable band gap of 2.1 eV and a longer irradiation time of 180 minutes under UV light, achieved a lower degradation efficiency of 87%. The functionalized graphene oxide/ZnO catalyst showed a high efficiency of 94% but required a longer irradiation time of 100 minutes and a high-power HQ1-400 W/D lamp. Its slightly higher rate constant of 0.049 min^−1^ suggests a faster reaction but at the cost of a longer operational time and a more powerful light source. The TiO_2_–Bi_2_O_3_–CuO catalyst supported on natural zeolite also demonstrated high efficiency (94.1%) but under a considerably longer irradiation time of 240 minutes and a relatively low rate-constant of 0.0062 min^−1^. This analysis confirms the effectiveness of the currently proposed photocatalytic system for the efficient removal of Safranin O dye from aqueous solutions.

**Table 4:** Performance comparison of rGO-GN10 with reported photocatalysts for SAF degradation

Catalyst	Preparation methods	Wight	Irradiation time	Light source	Mechanism	Rate constant (min^−1^)	Band gab	Degradation efficiency	Key findings	Ref.
rGO-GN10	Ultrasonic bath	0.02	60	Sun simulator	*Z* scheme	0.0349	2.22	93.6%	rGO-g-C_3_N_4_-NiO integration boosts charge separation and visible light absorption	This work
Functionalized graphene oxide/ZnO	Solution route + calcination	0.02	100	HQ1-400 W/D lamp	N.A	0.049	3.10	94%	Optimal GO loading (0.09 wt%) maximizes performance	52
LaNiSbWO_4_-G-PANI	Sonochemical synthesis	0.1	180	500 W Xe lamp	Type II	N.A	1.75	84%	PANI modification reduced band gap (1.75 eV) and enhanced visible-light absorption. – GO improved electron–hole separation	53
ZnO–S-g-C_3_N_4_	Liquid exfoliation of S-g-C_3_N_4_ and ZnO in distilled water	0.1	180	UV light	*Z*-scheme	0.00989	2.1	87%	S-doping introduces sulfur into g-C_3_N_4_ lattice, improving conductivity and charge separation	54
ZnO/CdS	Sol–gel synthesis	0.5	120	21-Watt LED cool daylight visible lamp	Type II	0.00304	2.46	100	Coupling CdS with ZnO reduced the bandgap from 3.30 eV to 2.46 eV, enabling visible light activation	55
TiO_2_–Bi_2_O_3_–CuO supported on natural zeolite	Ceramic method + Wet impregnation under ultrasonic waves	0.04	240	Sunlight	n–p heterojunction	0.0062	1.66	94.1	Zeolite support improved adsorption capacity and charge separation. Low band gap (1.66 eV) enabled efficient visible-light absorption	56

## 4 Conclusion

This work establishes the NiO/g-C_3_N_4_/rGO hybrid as a paradigm-shifting material for integrated contaminant removal, where new mechanistic insights reveal its unparalleled functionality. Quantifiable interfacial electronic restructuring (Ni 2p_3/2_ shift: +2.37 eV; 65° Warburg angle) enables rGO-mediated *Z*-scheme operation, directing high-potential holes (+1.4 eV) toward SAF oxidative degradation while generating radicals – with evidence confirming sustained ˙OH/O – production (>85% contribution to degradation). The 23 mg g^−1^ adsorption capacity, governed by multi-mechanistic binding and 4.2× selectivity, functions not merely as capture but as a kinetic accelerator: preconcentration slashes radical diffusion distances, enabling ultrafast mineralization (*k* = 0.0316 min^−1^). Three factors correlated structure-optics-performance enhancement of the ternary composite compared to its individual components; namely, (i) marginally increased visible absorbance and narrowed *E*_g_ to 2.19 eV; (ii) a 92% quenching of PL and 79% decrease of *R*_ct_ signals is correlated with high SAF removal rate; (iii) a 3.5× increase in BET surface area is correlated with faster adsorption and overall dye removal efficiency. Crucially, we discovered the composite's resilience in challenging environments, maintaining >90% efficiency in high-salinity (0.1 M NaCl) conditions. Thermodynamics confirm the process is exothermic (Δ*H* = −28.6 kJ mol^−1^) and entropy-driven, while pseudo-first-order kinetics dominate. Additionally, the ultrasound-assisted synthesis of the rGO-GN10 nanocomposite is both scalable and environmentally benign, from a practical standpoint. The aqueous-based, low-temperature method is inherently suitable for scale-up, avoiding toxic solvents and high energy costs. Furthermore, the composite's exceptional reusability and stable performance under visible light significantly reduce the long-term environmental footprint and operational costs, positioning it as a highly promising and sustainable candidate for real-world industrial wastewater remediation.

## Conflicts of interest

There is no conflicts to declare.

## Supplementary Material

RA-015-D5RA07330H-s001

## Data Availability

All data supporting the findings of this study are included in the main manuscript and the supplementary information (SI). Supplementary information is available. See DOI: https://doi.org/10.1039/d5ra07330h.
