# Supplementary material for: Synergistic adsorption-photocatalysis via rGO-mediated electron shuttling in ultrasonically synthesized NiO/g-C3N4-based ternary nanocomposites for Safranin O removal
Source: RSC Adv. 2025 Dec 3;15(55):47520–44. doi: 10.1039/d5ra07330h (PMC12679431; doi:10.1039/d5ra07330h)
Supplement: RA-015-D5RA07330H-s001 [file RA-015-D5RA07330H-s001.pdf]

Supplementary Information (SI) for RSC Advances.
This journal is © The Royal Society of Chemistry 2025

Synergistic Adsorption-Photocatalysis via rGO-Mediated Electron Shuttling in Ultrasonically Synthesized NiO/g-C_3N_4-Based Ternary Composites for Safranin O Removal

Mahmoud A. Ahmed [a,b*], Arafat Toghan [c*], Mohamed A. Ahmed [b], Ashraf A. Mohamed [b]

[a] Veolia Water Technologies, Cairo, 11835, Egypt
[b] Chemistry Department, Faculty of Science, Ain Shams University, Cairo-11566, Egypt
[c] Chemistry Department, College of Science, Imam Mohammad Ibn Saud Islamic University (IMSIU), Riyadh 11623, Saudi Arabia

*Corresponding author:

Mahmoud. A. Ahmed (mahmoudmahmoud_p@sci.asu.edu.eg)

Materials characterization

Fourier-transform infrared (FTIR) spectra were acquired using a KBr disk technique on an ATI Mattson FTIR spectrometer. High-resolution transmission electron microscopy (HRTEM) was employed to capture detailed images, utilizing a JEOl-2100-HRTEM instrument. X-ray photoelectron spectra were recorded using a K-ALPHA-XPS instrument (Themo Fisher Scientific, USA) with monochromatic X-ray Al Kα radiation ranging from 10 to 1350 eV. Diffuse reflectance spectroscopy measurements were conducted using a V-570-JASCO spectrometer. The FLS1000 Photoluminescence Spectrometer, developed by Edinburgh Instruments Ltd., United Kingdom, was used for recording PL spectra for accurate characterization of photoluminescent materials and precise measurement of their lifetime decay. Absorption measurements were performed in 10 mm matched cuvettes using a Shimadzu-1650 UV/VIS absorption spectrophotometer (Kyoto, Japan) controlled by UV-probe 2.5 software. The photocatalytic activity of the synthesized catalyst was evaluated by monitoring the photodegradation of SAF dye under a solar simulator operated with a 300W-Xe lamp and a UV cut-off filter.

Adsorption performance

The adsorption performance of the rGO-NiO/g-C_3N_4 hybrid was evaluated for the removal of Safranin O dye from aqueous solutions. In a typical experiment, 0.02 g of the hybrid catalyst was dispersed into 100 mL of Safranin O solution with varying initial concentrations (3.5×10^{-5} M to 1×10^{-5} M). The mixture was continuously agitated on a

magnetic stirrer at a fixed stirring speed (350 rpm) for 1 hour under ambient conditions. At predetermined time intervals (e.g., 10, 20, 30, 45, 60 min), aliquots were withdrawn and immediately centrifuged (6000 rpm, 10 min) to separate the catalyst particles. The residual Safranin O concentration in the supernatant was analyzed using a UV-Vis spectrophotometer by measuring the absorbance at its maximum absorption wavelength (λ_max = 520 nm).

The adsorption capacity (q_e, mg/g) were calculated using the following equations:

$$q_e = \frac{(C_o - C_e) * V}{m}$$

Where **C_0** represents the initial dye concentration (mg/L or M), **C_e** is the equilibrium dye concentration (mg/L or M), **C_t** denotes the dye concentration at time *t* (mg/L or M), **V** is the volume of the solution (L), and **m** stands for the mass of the adsorbent (g).

Adsorption Isotherm Analysis

To elucidate the equilibrium adsorption behavior of Safranin O onto the rGO-NiO/g-C_3N_4 hybrid, nonlinear regression was employed to fit experimental data to four isotherm models: the two-parameter Langmuir (monolayer adsorption), Freundlich (heterogeneous surface), and Temkin (adsorbate-adsorbent interactions) models, along with the three-parameter Sips model (hybrid Langmuir-Freundlich). Experiments were systematically conducted at four temperatures (15, 20, 25, and 30 °C) across a dye concentration range of 3.5×10^{-5} M to 1×10^{-5} M, while maintaining constant pH, adsorbent dosage (0.02 g/100 mL), and agitation speed.

The Langmuir model was applied to assess maximum monolayer capacity (q_m) and affinity (b), while the Freundlich model quantified surface heterogeneity (n) and adsorption intensity (K_f). The Temkin model provided insights into heat of adsorption (B)

and adsorbent-adsorbate binding energy. The Sips model, integrating features of both Langmuir and Freundlich, was used to evaluate whether adsorption transitioned from monolayer to multilayer behavior at higher concentrations. Equilibrium data were fitted using OriginLab 2022 (nonlinear least-squares algorithm), with model validity assessed via adjusted R^2. Temperature-dependent trends in fitted parameters (e.g., q_m, K_f, b) were analyzed to infer thermodynamic spontaneity and adsorption mechanisms. The Sips model's third parameter (n_S), which deviates from unity, confirmed whether adsorption deviated from ideal Langmuir behavior due to surface heterogeneity or cooperative effects.

Photocatalytic performance

To study the degradation of SAF on the surfaces of pristine g-C3N4, GN10, and rGO-GN10 composite materials, a solar simulator was used. **The solar simulator was calibrated to the AM 1.5G standard spectrum, and the light intensity was adjusted to 100 mW/cm² (1 sun) using a calibrated silicon photodiode meter.** The experimental procedure involved dispersing the photocatalyst in a glass reactor containing 100 mL of a 4×10^{-5} mol/L SAF solution. The mixture was stirred at 500 rpm in a dark environment for 1.0 hour to establish an equilibrium state of adsorption and desorption. Subsequently, the mixture was exposed to light from the solar simulator **for 2 hours**. At different time intervals, small sample aliquots (4 mL each) were taken from the solution mixture. These samples were then centrifuged and analyzed using UV-visible spectrophotometry. This allowed for the measurement of the light absorbance of the dye molecules. To identify the specific reactive species involved in photocatalytic activity, various free radical scavengers were employed. Isopropanol was used for •OH radical detection, ammonium oxalate for holes (h+) detection, and benzoquinone for superoxide radical detection. The assessment of terephthalic acid photoluminescence at 425 nm was conducted to estimate the production of hydroxyl radicals.
